# Metal Ruthenium Complexes Treat Spinal Cord Injury By Alleviating Oxidative Stress Through Interaction With Antioxidant 1 Copper Chaperone Protein

**DOI:** 10.1002/advs.202407225

**Published:** 2024-10-16

**Authors:** Juanjuan Li, Cheng Peng, Caiqiang Huang, Li Wan, Ke Wang, Ping Wu, Tianjun Chen, Guodong Sun, Rui Guo, Hongsheng Lin, Zhisheng Ji

**Affiliations:** ^1^ Department of Orthopedics The First Affiliated Hospital Jinan University Guangzhou Guangdong 510632 China; ^2^ Department of Urology Guangzhou Institute of Urology Guangdong Key Laboratory of Urology the State Key Laboratory of Respiratory Disease the First Affiliated Hospital of Guangzhou Medical University Guangzhou Medical University Guangzhou Guangdong 510230 China; ^3^ Guangdong Provincial Key Laboratory of Spine and Spinal Cord Reconstruction The Fifth Affiliated Hospital (Heyuan Shenhe People's Hospital) Jinan University Heyuan 517000 China; ^4^ Key Laboratory of Biomaterials of Guangdong Higher Education Institutes Guangdong Provincial Engineering and Technological Research Center for Drug Carrier Development Department of Biomedical Engineering Jinan University Guangzhou 510632 China

**Keywords:** antioxidant 1 copper chaperone, reactive oxygen species, ruthenium metal complexes, spinal cord injury

## Abstract

Oxidative stress is a major factor affecting spinal cord injury (SCI) prognosis. A ruthenium metal complex can aid in treating SCI by scavenging reactive oxygen species via a protein‐regulated mechanism to alleviate oxidative stress. This study aimed to introduce a pioneering strategy for SCI treatment by designing two novel half‐sandwich ruthenium (II) complexes containing diverse N^N‐chelating ligands. The general formula is [(η^6^‐Arene)Ru(N^N)Cl]PF_6_, where arene is either 2‐phenylethanol‐1‐ol (bz‐EA) or 3‐phenylpropanol‐1‐ol (bz‐PA), and the N^N‐chelating ligands are fluorine‐based imino‐pyridyl ligands. This study shows that these ruthenium metal complexes protect neurons by scavenging reactive oxygen species. Notably, η6‐Arene substitution from bz‐PA to bz‐EA significantly enhances reactive oxygen species scavenging ability and neuroprotective effect. Additionally, molecular dynamics simulations indicate that the ruthenium metal complex increases Antioxidant 1 Copper Chaperone protein expression, reduces oxidative stress, and protects neurons during SCI treatment. Furthermore, ruthenium metal complex protected spinal cord neurons and stimulated their regeneration, which improves electrical signals and motor functions in mice with SCI. Thus, this treatment strategy using ruthenium metal complexes can be a new therapeutic approach for the efficient treatment of SCI.

## Graphical abstract

This study designs two half‐sandwich ruthenium (II) complexes that protect neurons by scavenging ROS. Notably, the substitution of the η6‐arene ligand from bz‐PA to bz‐EA significantly enhances the ROS scavenging ability and the neuroprotective effect. Additionally, the metal complex with bz‐EA modulates the expression of ATOX1, thereby reducing oxidative stress and protecting neurons, which enhances the therapeutic efficacy for SCI.

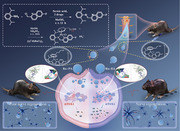

## 1 Introduction

Spinal cord injury (SCI) is a common and serious neurological condition that typically results from traumatic injury.^[^ ^1^, ^2^, ^3^ ^]^ Spinal cord damage leads to loss of control over the higher nerve centers, which results in complete or partial loss of sensory and motor functions below the injury site and causes severe paralysis or death.^[^ ^4^, ^5^, ^6^ ^]^ According to the World Health Organization, the global incidence of SCI ranges from 3.6–195.4 per million people per year, with most patients being young adults. This places a significant burden on their families.^[^ ^7^, ^8^, ^9^ ^]^

Secondary injury to SCI is a multipathological process that follows primary injury and encompasses a range of biochemical responses, including oxidative stress, inflammatory responses, and lipid peroxidation.^[^ ^10^, ^11^ ^]^ This process generates numerous reactive oxygen species (ROS), including superoxide anions and monoclinic oxygen.^[^ ^12^, ^13^ ^]^ These substances initiate reactions that damage the subcellular structures of the neurons and glial cells, leading to cell death. This may be the primary reason for poor prognosis in patients with SCI.^[^ ^14^, ^15^ ^]^ The current focus is on effectively removing ROS, alleviating inflammation, and reducing neuronal death.^[^ ^16^, ^17^, ^18^, ^19^ ^]^ Consequently, the development of effective therapeutic strategies for secondary SCIs may accelerate patient recovery.

The neutralization of excess ROS from inflammatory cells substantially reduces secondary injuries associated with acute SCI. The antioxidant 1 copper chaperone (ATOX1) protein facilitates the transfer of copper ions to copper‐dependent antioxidant enzymes such as superoxide dismutase (SOD1).^[^ ^13^ ^]^ SOD1 activation by ATOX1 indirectly strengthens cellular antioxidant defense. Furthermore, it protects cells by modulating intracellular copper ion levels, which during overabundance generate harmful free radicals and induce oxidative stress. Hence, the development of ATOX1‐targeting drugs is crucial for treating neurological disorders.^[^ ^20^, ^21^ ^]^ In addition, activated by copper, Atox1 acts as an innovative transcription factor that facilitates nuclear translocation, DNA binding, and transactivation, which in turn promotes cell proliferation.^[^ ^22^, ^23^ ^]^

Research on the treatment of neurological diseases is continuously expanding, covering a variety of cutting‐edge fields, including: CREB phosphorylation is required for neurotrophin expression,^[^ ^24^ ^]^ which is of key importance for preventing and regenerating neurological disorders, nanoparticle‐mediated drug‐delivery systems,^[^ ^25^ ^]^ liquid crystalline lipid nanoparticles^[^ ^26^ ^]^ and so on. Meanwhile, Polydopamine nanoparticles are notable for their biodegradability and biocompatibility,^[^ ^27^ ^]^ but ruthenium‐based compounds also exhibit these important qualities and are significant in biomedicine. The semi‐sandwich structure of ruthenium metal complexes provides unique electronic properties that excel in antioxidant capabilities, contributing to their superior performance. Additionally, these ruthenium complexes show improved stability in biological systems, a key factor for sustaining their therapeutic effects throughout treatment. The ubiquity of heterocyclic structures and heteroatoms in pharmaceuticals underscores their importance in drug discovery, and functionalizing these elements is a key step. More than 20% of contemporary pharmaceuticals contain fluorine atoms. Although nascent, the metalation of heterocyclic systems such as naphthyridines has revealed their potential to treat a spectrum of diseases, making them prime candidates for future medicinal chemistry.^[^ ^28^, ^29^ ^]^

**Scheme 1:**
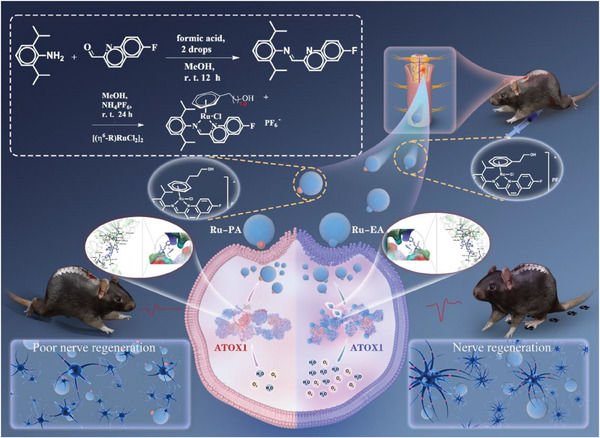
Interaction of metal‐ruthenium complexes with ATOX1 protein to alleviate oxidative stress for the treatment of spinal cord injury.

This study introduces a pioneering strategy that combines fluorine incorporation with advanced bioimaging to create two novel ruthenium (II) half‐sandwich complexes. These complexes are characterized by diverse N^N‐chelating ligands, represented by the formula [(η6‐Arene)Ru(N^N)Cl]PF_6_, where the N^N ligands are fluorine‐enriched imino‐pyridyl types. The arenes 2‐phenylethanol‐1‐ol (bz‐EA) and 3‐phenylpropanol‐1‐ol (bz‐PA) were used to yield Ru‐EA and Ru‐PA complexes. Replacing the bz‐PA η^6^‐Arene with bz‐EA dramatically boosts the ROS neutralizing ability and neuroprotective efficacy of the complexes. Molecular dynamics simulations have shown that the ruthenium complex bz‐EA stimulates ATOX1 production, which counters oxidative stress and protects neurons in SCI scenarios. Furthermore, the ruthenium metal complex Ru‐EA preserved neurons and encouraged their regrowth, which improved electrical conduction and motor capabilities in spinal cord‐injured mice. This therapeutic intervention using ruthenium metal complexes opens new possibilities for the effective management of SCIs (**Scheme** **1**).

## 2 Results and Discussion

### 2.1 Characterization of Drugs

**Figure 1:**
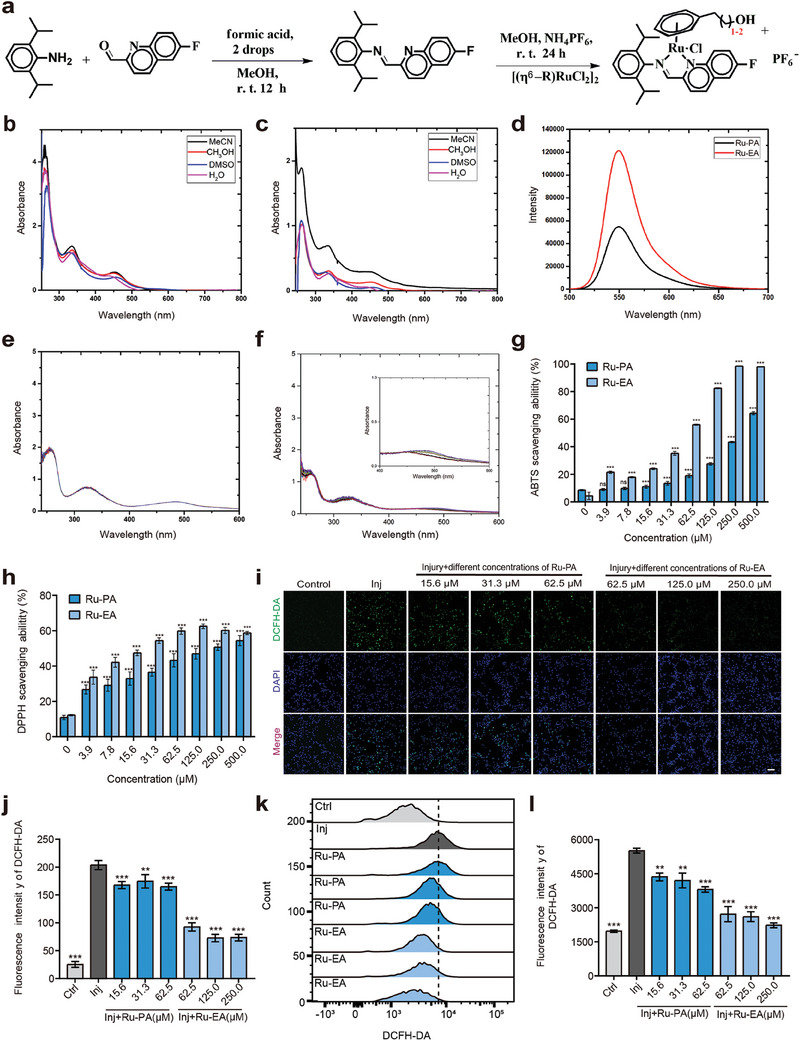
Synthesis and characterization of Ru‐PA and Ru‐EA and in vitro ROS scavenging effects. a) Steps for Ru‐EA and Ru‐PA synthesis. Electronic absorption spectra of Ru‐EA b) and Ru‐PA c) complexes in solvents such as water, methanol, dimethylsulfoxide, and acetonitrile at 298 K. d) Fluorescence spectra of Ru‐EA and Ru‐PA complexes at 298 K. The hydrolytic stability of Ru‐EA e) and Ru‐PA f) complexes in DMSO/H_2_O solution (2:8 ratio) monitored using UV–vis spectroscopy. Scavenging capacity of ABTS g) and DPPH h); free radicals at different drug concentrations in 2 h (n = 3, repeated measurement one‐way ANOVA). i) Representative plots of intracellular ROS levels of HT22 cells detected using flow cytometric assays. j) Statistical plot of high and low intracellular ROS levels (n = 3, repeated measurement one‐way ANOVA). k) Representative fluorescence plots of intracellular ROS levels in each group of cells captured using confocal microscopy. l) Statistical plot of high and low intracellular ROS levels (n = 5, repeated measurement one‐way ANOVA). **p < 0.01, ***p < 0.001; n represents the number of samples in each group; Ctrl represents the control group, and Inj represents the injury‐alone group.

Ruthenium dimers [(η^6^‐arene)RuCl_2_]_2_ were formed by allowing a series of 2,5‐dihydrophenyl‐substituted alcohols to react with RuCl_3_ under reflux in absolute ethanol. Iminopyridyl ligands were synthesized through condensation processes, which yielded satisfactory results. Two distinct half‐sandwich metal complexes featuring diverse N^N‐chelating ligands with the general formula [(η^6^‐Arene)Ru(N^N)Cl]PF_6_ were prepared. The arenes in these complexes were 2‐phenylethanol‐1‐ol (bz‐EA) and 3‐phenylpropanol‐1‐ol (bz‐PA), and the N^N‐chelating ligands were fluorine‐substituted iminopyridines. These complexes were synthesized through the interaction of the ligands with ruthenium dimer precursors in methanol at a standard temperature. The synthesis route and preparation of the half‐sandwich Ru II complexes are outlined in **Figure** **1**a. The structural formulae of Ru‐PA and Ru‐EA are shown in Figure S1 (Supporting Information). These complexes were characterized using ^1^H NMR spectroscopy and elemental analysis, and all complexes were isolated in the form of hexafluorophosphate (PF_6_^−^) salts. The electronic absorption spectra of the Ru‐EA and Ru‐PA complexes in water, methanol, dimethyl sulfoxide, and acetonitrile at room temperature (298 K) are illustrated in Figure 1b,c. The prominent absorption bands below 300 nm may be attributed to the spin‐allowed ligand‐centered π‐π* transitions. The featureless bands within the 300–360 nm range may be attributed to the phenyl‐to‐pyridine π‐π* ligand‐centered charge transfer and metal‐to‐ligand charge transfer (MLCT) transitions that are represented by dπ(Ru) to π*(phenyl) transitions. The bands in the visible spectrum (>360 nm) correspond with both singlet and triplet MLCT transitions. Minor variations in the Ru‐EA absorption spectra were discernible in solvents with different polarities, whereas significant differences were observed in the Ru‐PA spectra across these polarities. The fluorescence spectra of Ru‐EA and Ru‐PA complexes at room temperature are shown in Figure 1d. Both complexes emitted a blue‐green glow with a peak wavelength of ≈550 nm upon excitation at 405 nm; Ru‐PA exhibited a more pronounced fluorescence intensity than Ru‐EA. The hydrolysis of M─Cl bonds by water is typically an essential activation step for anticancer complexes based on transition metals. The hydrolytic stabilities of the Ru‐EA and Ru‐PA complexes in a DMSO/H_2_O solution (2:8 ratio) were monitored using UV–vis spectroscopy (Figure 1e,f). The data showed that the stability of Ru‐EA was superior to that of the Ru‐PA complex.

The ABTS [2,2′‐azinobis‐(3‐ethyl‐benzothiazoline‐6‐sulfonate)] radical scavenging method, which was used to test the antioxidant capacity of Ru‐EA and Ru‐PA.^[^ ^30^ ^]^ show that the absorbance of the solution gradually decreased as Ru‐EA and Ru‐PA concentrations increased over time; however, the decrease was more pronounced in the Ru‐EA group. This indicates that both Ru‐EA and Ru‐PA possess strong antioxidant capacities, although Ru‐EA was more potent than Ru‐PA (Figure 1g). DPPH free radical scavenging experiments were performed to enhance the reliability of these results, and similar outcomes were obtained (Figure 1h).

### 2.2 Intracellular Antioxidant Capacity of Drugs

Ru‐EA and Ru‐PA possess strong antioxidant properties. The HT22 mouse hippocampal neuron cell line was used for the in vitro study to determine their ROS scavenging ability to achieve cellular antioxidant effect and compare the strength of their effects. HT22 cells are highly sensitive to glutamate and produce large quantities of ROS following glutamate‐induced injury; hence, this is an effective model for studying oxidative stress in vitro.^[^ ^31^, ^32^ ^]^ To determine the optimal working concentrations of the two drugs, the CCK‐8 kit was used to test the toxicity of different drug concentrations in HT22 cells. As shown in Figure S2 (Supporting Information), a series of complete culture media containing the two drugs at different concentrations were prepared using the half‐volume dilution method. Ru‐PA elicited higher toxicity in HT22 cells than Ru‐EA. Based on the results, the highest working concentrations for Ru‐EA and Ru‐PA for subsequent experiments were set at 250.0 and 62.5 µM, respectively.

DCFH‐DA and DHE kits were used to detect the intracellular ROS levels in the HT22 cells to observe the antioxidant effect elicited by the two drugs. The DCFH‐DA kit used the fluorescent probe DCFH‐DA to detect the total ROS level. DCFH‐DA enters the cells through the cellular membrane and is hydrolyzed by esterases to produce DCFH, which cannot penetrate the cell membrane, leading to its retention within the cell. Intracellular ROS then convert non‐fluorescent DCFH into DCF, which emits green fluorescence. Therefore, the level of intracellular DCF green fluorescence reflects the total intracellular ROS level.^[^ ^33^, ^34^ ^]^ The results showed that intracellular green fluorescence intensity increased significantly after the treatment of HT22 cells with glutamate, indicating a state of strong oxidative stress (Figure 1i,j). Treatment with Ru‐EA and Ru‐PA decreased the fluorescence intensity compared with that of the pure glutamate damage group; this reduction was more pronounced in the Ru‐EA group than in the Ru‐PA group. This indicates that both drugs reduce intracellular ROS levels to elicit an antioxidant effect; however, Ru‐EA is more potent than Ru‐PA.

To validate these experimental results, flow cytometry was used to test the ability of the drugs to reduce intracellular ROS levels. These results were similar to those of previous results that Ru‐EA significantly reduces intracellular ROS levels in HT22 cells and was more effective than Ru‐PA (Figure 1k,l).

As the superoxide anion is the precursor of many intracellular ROS, detecting the superoxide anion levels better reflects cellular oxidative stress.^[^ ^13^ ^]^ DHE is a widely used superoxide anion fluorescence detector that can be ingested by living cells and reacts with intracellular superoxide anions to form products such as ethidium bromide. These products bind to the RNA or DNA and emit red fluorescence. Increase in the intracellular superoxide anion level increases ethidium bromide production, resulting in stronger red fluorescence.^[^ ^35^ ^]^ Treating HT22 cells at the oxidative stress state with different drug concentrations significantly reduced cellular red fluorescence intensity compared with that of the untreated subgroups. This result is similar to that observed during the DCFH‐DA probe experiments, suggesting that both drugs are effective in scavenging superoxide anions and reducing intracellular ROS levels, although Ru‐EA is more effective than Ru‐PA (Figure S3a,b, Supporting Information). The results also showed that the intracellular scavenging ability of Ru‐EA for ROS was much better than that of MnTBAP. To understand exactly what kind of ROS Ru‐PA and Ru‐EA clears, we used the Singlet Oxygen Sensor Green (SOSG) fluorescent probe to detect the levels of singlet oxygen (^1^O_2_). SOSG is a highly selective fluorescent probe for singlet oxygen, and it is very useful in detecting the levels of singlet oxygen. The results indicate that the complex Ru‐EA can significantly reduce the levels of singlet oxygen. Meanwhile, we used a hydrogen peroxide assay kit to determine the concentration of hydrogen peroxide in HT22 cells by oxidizing divalent iron ions to produce trivalent iron ions, which then formed a purple product with xylene orange in a specific solution. The results showed that the level of intracellular hydrogen peroxide decreased after the addition of Ru‐EA compared to the damaged group, indicating that Ru‐EA also has some scavenging ability for hydrogen peroxide (Figure S3c,d, Supporting Information). In summary, both Ru‐EA and Ru‐PA reduced the intracellular ROS levels in HT22 cells to elicit an antioxidant effect, and Ru‐EA showed the higher efficacy of the two drugs.

### 2.3 Protective Effects of Drugs on Neurons

Neurons are sensitive to peroxidized environments and exhibit low tolerance and susceptibility to oxidative stress and damage. Ru‐EA exhibits strong antioxidant effects.^[^ ^36^ ^]^ Therefore, Ru‐EA may have potential neuroprotective applications. Subsequent experiments were performed with extracted primary neurons to explore the oxidative stress alleviating effect exerted through ROS scavenging, which protects neurons and promotes neuronal repair after injury.

**Figure 2:**
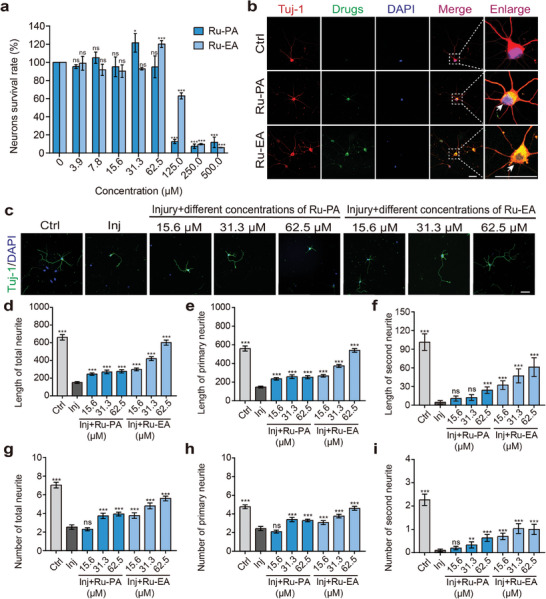
In vitro Ru‐PA and Ru‐EA distribution and neuroprotective activity in primary neurons. a) Detecting the effect of both drugs on survival of primary neurons (n = 3, repeated measurement one‐way ANOVA). b) Distribution of both drugs in primary neurons; the white dashed box shows an enlarged view; the arrow points to the neuronal cytosol. c) Representative plots of the neuronal protective effects of Ru‐PA and Ru‐EA. Statistical plots of total length d), primary protrusion length e), secondary protrusion length f), total number of protrusions g), number of primary protrusions h), and number of secondary protrusions i) of each group of primary neurons (n = 30, repeated measurement one‐way ANOVA). Scale bar = 50 µm; *p < 0.05, **p < 0.01, ***p < 0.001.

Similar to previous experiments, the CCK‐8 kit was used to test the toxicity of the drug in primary neurons, and the maximum working concentration was selected based on the experimental results (**Figure** **2**a). To explore drug uptake, the primary neurons were incubated with the drug at the maximum non‐toxic working concentration. The results showing green fluorescence indicate that both Ru‐EA and Ru‐PA were taken up by the neurons, and drugs aggregated in the cytosol and protruding parts of the neurons (Figure 2b).

The neuroprotective effects elicited by the drugs were observed by treating glutamate‐injured neurons with different concentrations of the drugs and observing and counting the lengths and number of first‐ and second‐order protrusions and total protrusions.^[^ ^37^ ^]^ As shown in Figure 2c–i, the glutamate‐lesioned neurons showed significantly reduced lengths and number of protrusions, which were recovered after treatment with these drugs. This trend became more pronounced as drug concentration increased, and the neuroprotective effect exerted by Ru‐EA was better than that of Ru‐PA. Meanwhile, the neuroprotective effect of Ru‐EA was superior to that of TEMPO and MnTBAP at non‐toxic concentrations.

### 2.4 In Vivo Effects of Drugs to Alleviate Oxidative Stress and Inflammation

**Figure 3:**
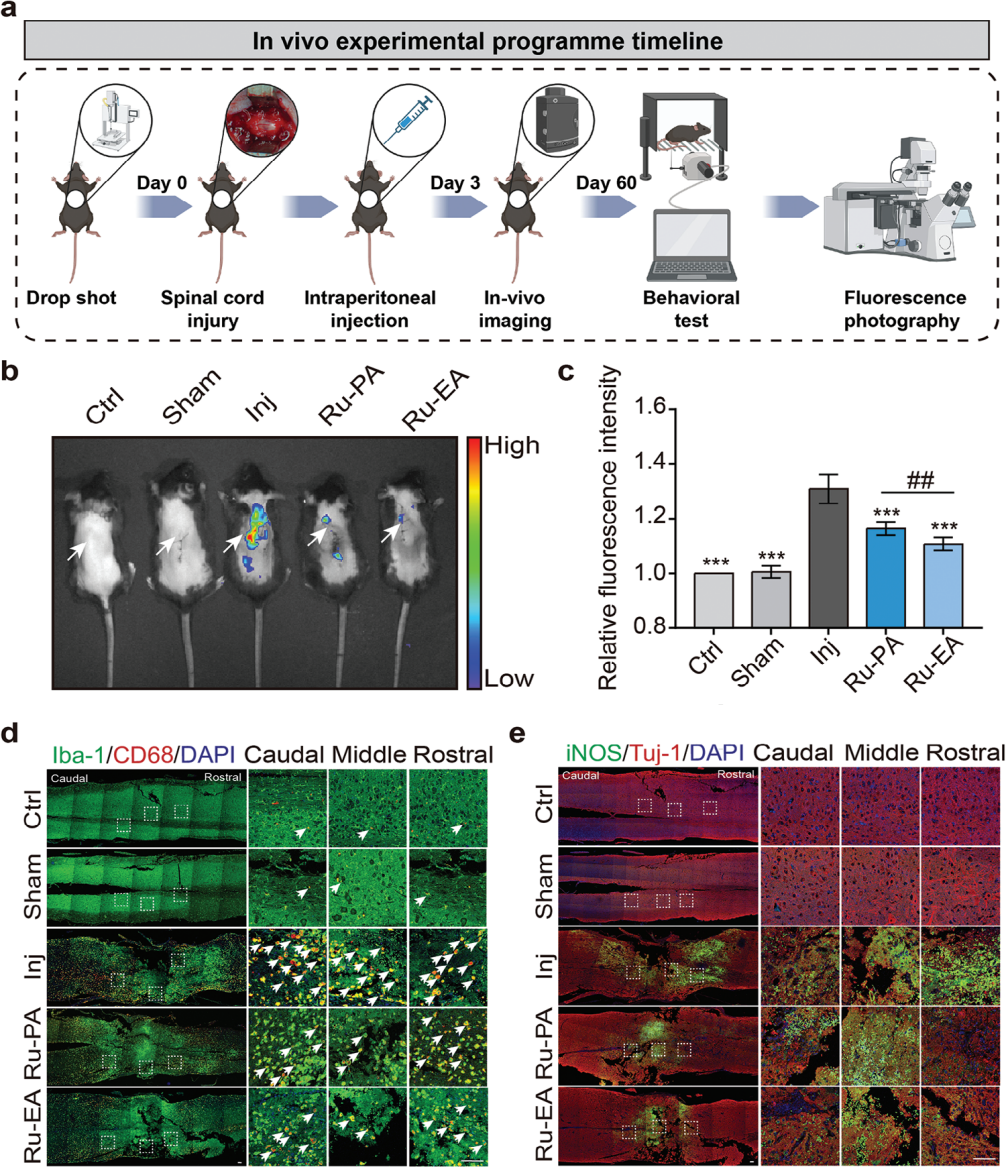
Ru‐PA and Ru‐EA alleviate ROS levels and inflammatory responses in mouse spinal cord at an early stage. a) Time flow diagram of in vivo mouse study. b) Representative images of oxidative stress levels detected in mouse spinal cord sites involved in L‐012; arrows points to the SCI site. c) Statistical plots of fluorescence intensity of oxidative stress in each group (mean ± SEM, n = 3, one‐way ANOVA). d) Representative maps of inflammation in Iba‐1‐ and CD68‐labeled mouse SCI segments; white dotted box shows magnified view in sequence from tail end to head end; arrows point to Iba‐1‐ and CD68‐positive cells. e) Representative map of inflammation in iNOS‐labeled mouse SCI segments. Scale bar = 100 µm; *p < 0.05, ^##^ *P* or **p < 0.01, ***p < 0.001.

A mouse spinal cord contusion model was established to explore the oxidative stress alleviating and neuroinflammation inhibiting effect of Ru‐EA in vivo. Neuronal protection and promotion of injured spinal cord recovery were elucidated by observing oxidative stress and inflammation levels. Oxidative stress levels are highest during the acute and subacute phases during week 1 of SCI; hence, controlling oxidative stress and inflammation during this stage promotes SCI repair.^[^ ^16^, ^18^ ^]^ Therefore, the mice with SCI were continuously administered the drug for 7 days after successful modeling, and the levels of oxidative stress and inflammation were tested 3 days after drug administration. A flowchart of the animal experiment is shown in **Figure** **3**a.

On day 3, L‐012 was injected into each group of mice to measure oxidative stress levels. L‐012 is a sensitive chemiluminescent probe that reacts with various types of reactive oxygen species. L‐012 is injected into animals prior to the start of the experiment, and oxidative stress levels are monitored through live imaging of small animals.^[^ ^38^, ^39^ ^]^ As shown in Figure 3b,c, the Ru‐EA‐treated mice showed lowered fluorescence intensity in the spinal cord compared with that of the untreated group. Similarly, Ru‐PA treatment produced a small degree of reduction in fluorescence intensity, suggesting that it removes ROS in mice and alleviates oxidative stress.

Iba‐1 is highly expressed in microglia and is involved in inflammatory responses. During neurological injury, microglia proliferate, migrate, and activate Iba‐1 expression, which contributes to inflammatory response.^[^ ^40^ ^]^ CD68 is a glycoprotein that is primarily expressed on the surface of monocytes, macrophages, and other cells. During inflammatory response, CD68‐positive monocytes and macrophages migrate to the inflammation sites. Thus, changes in CD68 expression levels reflect the activation status of these cells and their degree of involvement in inflammatory response.^[^ ^41^ ^]^ Spinal cord tissues were obtained from mice on day 3 after SCI and the level of neuroinflammation was assessed by labeling the tissues with Iba‐1 and CD68 for immunofluorescence staining. As shown in Figure 3d, the spinal cord tissues of mice in the injury‐only group contained a large number of Iba‐1‐ and CD68‐positive cells in the injured area, indicating strong inflammatory response. In contrast, the Ru‐EA‐treated group showed significantly reduced levels, indicating that Ru‐EA treatment alleviates neuroinflammation at an early stage.

iNOS is an enzyme that is induced by cells in response to specific stimuli. During inflammation, iNOS overexpression and NO overproduction may lead to tissue damage and increased inflammation.^[^ ^42^ ^]^ Therefore, the results were validated by performing iNOS labeling for immunofluorescence staining and observation. The results show that iNOS expression was significantly reduced at the spinal cord injury site in the Ru‐EA‐treated group, suggesting that Ru‐EA alleviated neuroinflammation (Figure 3e). This concurs with the previous findings. In summary, Ru‐EA treatment significantly alleviates oxidative stress and inflammatory response in the spinal cord of SCI mice at an early stage and is more effective than Ru‐PA.

### 2.5 Effects of Drugs on Motor Function Recovery in SCI Mice

As Ru‐EA effectively alleviates oxidative stress and inhibits neuroinflammation in mice, behavioral experiments were performed to ascertain if it promotes the recovery of lower limb motor function in SCI mice.

**Figure 4:**
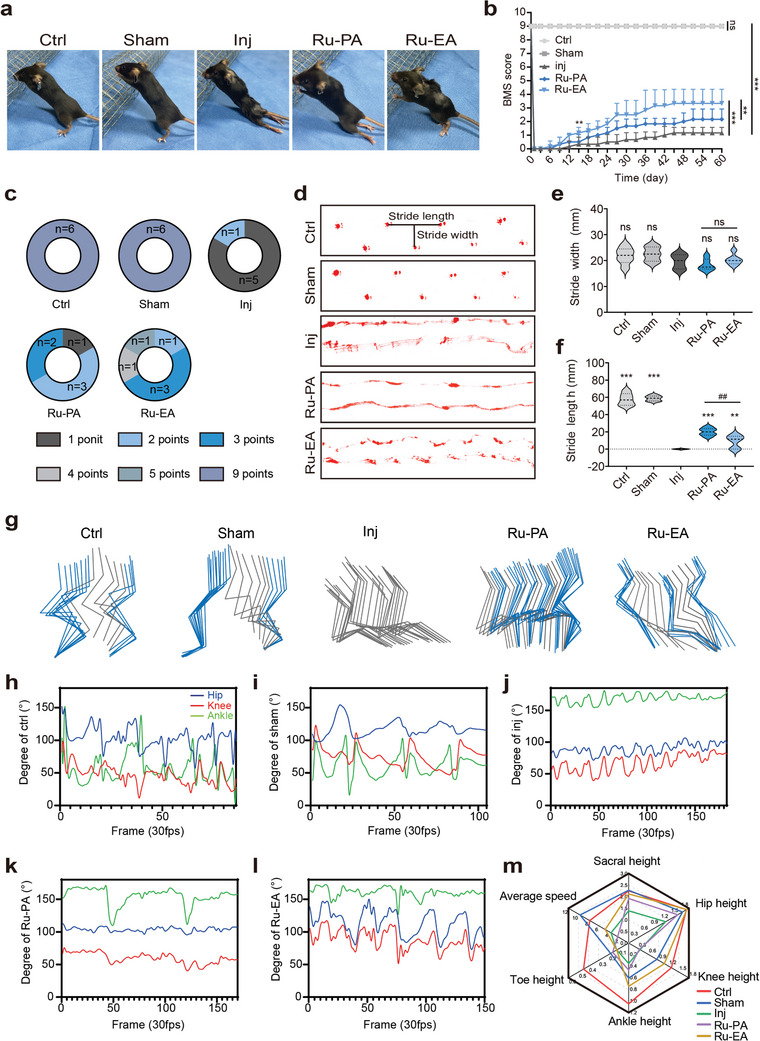
Effects of Ru‐PA and Ru‐EA on motor functions of both hind limbs in mice. a) Representative plots of hindlimb recovery at day 60. b) Statistical plots of BMS scores (n = 6, two‐way ANOVA). c) Pie chart of BMS score (Total = 6). d) Representative maps of mouse footprints for each group. Statistical plots of mouse (n = 6, one‐way ANOVA) step width e) and step length f). g) Changes in hip, knee, and ankle joint angles during crawling in ctrl h), sham i), inj j), Ru‐PA k), and RU‐EA l) mouse groups. m) Radar plots of mean sacral, hip, ankle, and toe heights and mean speeds during locomotion in each mouse group. ^##^ *P* or **p < 0.01, ***p < 0.001.

The Basso Mice Scale (BMS) score is a reliable indicator of hind limb ankle joint mobility, coordination, and paw posture and was used to evaluate changes in hind limb motor function in SCI mice.^[^ ^43^ ^]^ The movements of both lower limbs were timed in each group every 3 days, and the BMS scoring method was used to assign scores. **Figure** **4**a,b show gross images of the hind limbs on day 60 after modeling and the BMS scores for 60 days. The control and sham groups showed unrestricted motor function with a sustained BMS score of 9, whereas the BMS scores were reduced to 0 after successful modeling in the injury and drug‐treated groups (Figure 4b). The scores gradually increased over time, with a statistically significant difference between the scores of the injury and Ru‐EA groups on day 15; the Ru‐EA group showed a significantly higher score than that of the injury group at approximately day 60. This trend continued until the plateau phase. The Ru‐PA group showed a significant difference between the injury and Ru‐PA group scores on day 21, and the final BMS score of the Ru‐PA group was lower than that of the Ru‐EA group. A pie chart of the per‐mouse scores for each group is shown in Figure 4c. In conclusion, Ru‐EA treatment significantly promotes the recovery of motor function in both lower limbs of mice with SCI, and the effect was much better than that of Ru‐PA.

Additionally, a footprint experiment was performed to analyze the movement of mouse lower limbs by allowing each group of mice to crawl freely on clean paper and recording their footprints. The results showed that the mice in the control and sham‐operated groups had regular gait, whereas those of the injury group showed dragging, and those of the drug‐treated group showed a certain degree of gait restoration compared with that of the injury group (Figure 4d–f). As shown in Figure S4 (Supporting Information), the inclined plate crawling experiment yielded similar results.

DeepLabCut is an efficient method for 3D unlabeled pose estimation based on deep neural network transfer learning, which requires very little training data to obtain excellent results. This was used to map the shape and trajectory of both hindlimbs during mouse locomotion.^[^ ^44^, ^45^, ^46^ ^]^ Figure 4g shows a typical plot of the number of keyframes of the intercepted hind limb movement trajectories for each mouse group during exercise. The knee and ankle joints of the hind limbs of the injury group mice were stiff and continued to drag. The joints of the Ru‐PA group mice showed a certain degree of mobility, whereas those of the Ru‐EA group mice showed substantial increase in mobility with a stride‐walking posture. This indicates that the Ru‐EA group mice showed the best recovery of hind limb locomotor function. Figure 4h–l show the curves of the changes in the angle of each joint during exercise for each mouse group. Additionally, the average height and average velocity of each mouse joint was analyzed statistically, and the results are shown in Figure 4m. The joint heights in the Ru‐EA treatment group were higher than those of the Ru‐PA and injury‐only groups, suggesting that Ru‐EA treatment group mice stopped crawling with a dragging posture after treatment and exhibited a certain degree of normal gait.

### 2.6 Effects of Drugs on the Injured Spinal Cord in Mice

**Figure 5:**
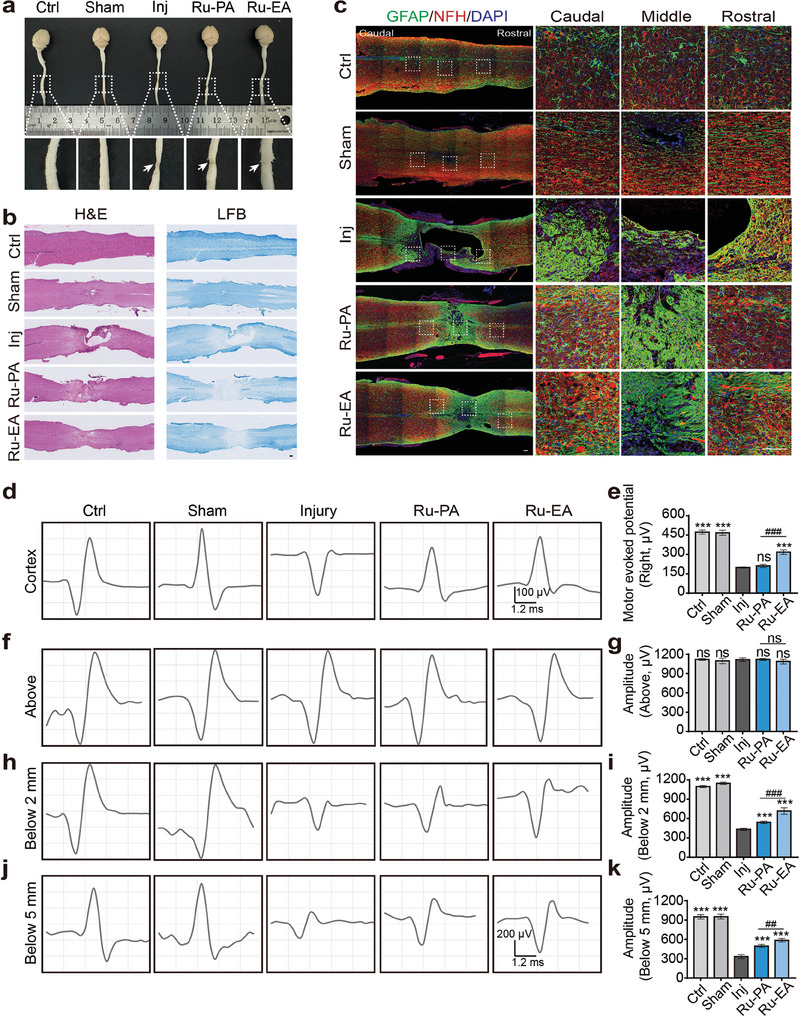
Effects of Ru‐PA and Ru‐EA on spinal cord tissue and electrical conduction recovery in mice. a) General view of the spinal cord tissue magnified in the white dashed box; the arrows point to the SCI site. b) Representative images of H&E and LFB staining of the spinal cord. c) Representative diagrams of neural recovery in GFAP‐ and NFH‐labeled mice; white dashed boxes show zoomed views in sequence from caudal to cephalic ends. Representative images of waveforms collected from electrophysiological experiments stimulating the right cerebral cortex of mice d) and statistical plots of wave amplitudes e). Representative images of waveforms collected during stimulation of the areas proximal f,g), distal 2 mm h,i), and distal 5 mm j,k) of the SCI site in mice and statistical plots of wave amplitudes (mean ± SEM, n = 6, one‐way ANOVA). Scale bar = 100 µm; ^##^ *p* < 0.01, ^###^ *p* or ***p < 0.001.

Spinal cord tissue was obtained from mice for pathological investigation to examine the impact of medication therapy following SCI. First, visual inspection of the removed spinal cords showed clear cavities from the lesion in the injury group, whereas those of the drug‐treated group showed fewer cavities and better integrity. This difference was particularly noticeable in the Ru‐EA group (**Figure** **5**a). H&E staining revealed poor continuity of the spinal cord tissue in the injury group and better continuity in Ru‐EA‐treated mice. LFB staining of the spinal cord revealed the myelin structure of the nerves (Figure 5b), and the myelin structure was more regular in the Ru‐EA treatment group than in the other group.

Immunohistochemical analysis was performed with specific neuromarkers to further study the protective and reparative effects of these drugs on spinal cord tissues. Glial fibrillary acidic protein (GFAP) is primarily distributed in astrocytes in the CNS and is used to label astrocytes and glial scarring.^[^ ^47^ ^]^ Neurofilament protein‐H (NFH) is an intermediate filament in the cytoplasm of neurons and is used to label neurons in spinal cord tissues.^[^ ^48^ ^]^ The status of neurons and glial scarring after SCI was observed after co‐staining with GFAP and NFH (Figure 5c). Compared with that of the injury group, the Ru‐EA‐treated mice showed less dense glial scarring and more surviving neuronal cells in the spinal cord, suggesting that Ru‐EA may inhibit the production of glial scarring and increase the neuron survival.

### 2.7 Effects of Drugs on Electrophysiology in SCI Mice

SCI causes a large number of neurons with signaling functions to break and die at the injury site, which leads to weakened signaling. Neurophysiological tests show decreased motor‐evoked potential (MEP) and amplitude of spinal cord electrical conduction signals.^[^ ^49^ ^]^ Therefore, electrophysiological assays were performed to assess nerve recovery in the spinal cords of SCI mice. The cerebral cortex was stimulated with an electrode to receive electrical signals from the gastrocnemius muscle of the contralateral calf and record motor‐evoked potentials. The spinal cord was stimulated proximal to the SCI site using an electrode. The electrical signals were received proximal, 2 mm distal, and 5 mm distal to the SCI injury site.

The results indicated that the MEP amplitude was lower in of mice after SCI than in the control group mice, suggesting that the corticospinal tract was significantly damaged. However, after drug treatment, motor‐evoked potentials recovered significantly with Ru‐EA group mice showing more significant recovery. This indicates that Ru‐EA treatment significantly increased the number of neurons with electrical conduction functions in the anterior horn of the spinal cord (Figure 5d,e; Figure S5, Supporting Information). Additionally, stimulation of the spinal cord tissue proximal to the SCI site showed no significant change in the amplitude of the electrical signals above the injured segments, whereas that of the electrical signals received 2 mm distal to the SCI site was lower than that of the ctrl group, indicating significant neuronal damage. However, the amplitude increased to a certain extent after drug treatment in the Ru‐EA group showing a more significant increase than that of the Ru‐PA group, indicating that Ru‐EA promoted neuronal recovery. The amplitudes of the electrical signals 5 mm distal to the SCI site showed similar trend (Figure 5f–k). Thus, the electrophysiological results indicate that Ru‐EA treatment significantly promotes spinal cord signaling recovery in SCI mice.

### 2.8 Biocompatibility Assessment of Drugs

Further investigation of drug toxicity to mouse organs was performed by collecting sections of each organ on day 60 of the animal experiment and staining with H&E for observation. The results presented in Figure S6 (Supporting Information) did not show any pathological alterations in these organs, indicating that Ru‐EA and Ru‐PA are biocompatible in mice and do not exhibit obvious toxicity.

### 2.9 Proteomic Analysis of the Spinal Cord

**Figure 6:**
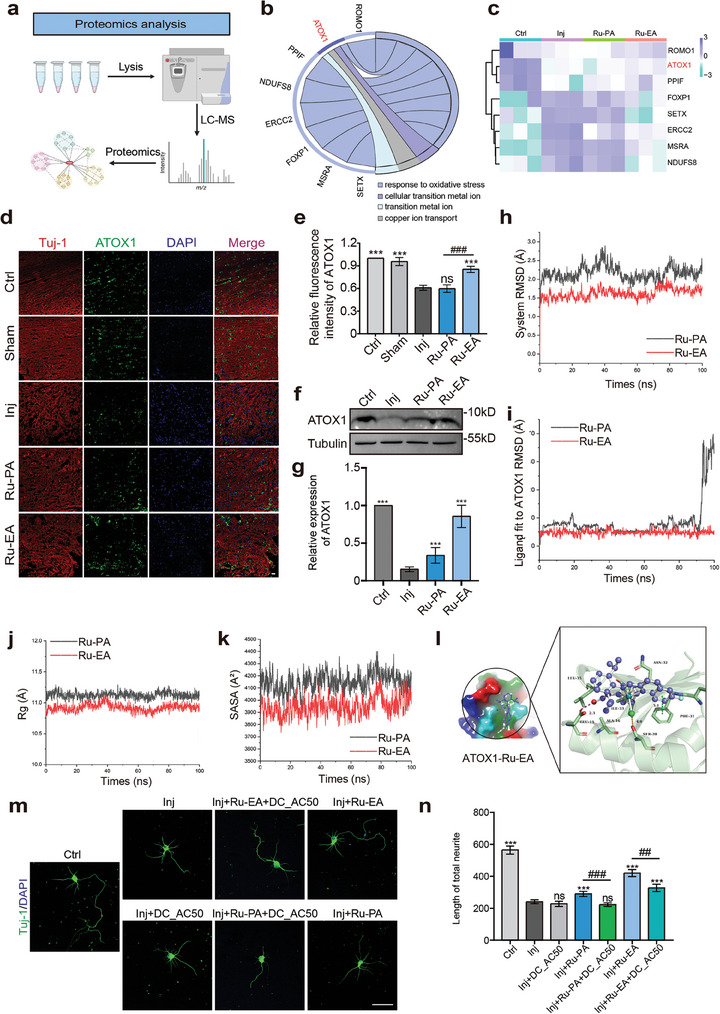
Proteomic study of Ru‐PA and Ru‐EA reveals interaction with ATOX1. a) Experimental flowchart for proteomics analysis. b) Circos diagram of key proteins analyzed using proteomics c) Heatmap of the expression of relevant proteins in each group. Fluorescence typical plots of changes in ATOX1 expression in mouse spinal cord tissues d), and statistical plots of relative fluorescence intensity of the protein e), mean ± SEM, n = 6, one‐way ANOVA). Typical plots of western blotting experiments f), and statistical plots of relative grey values of proteins g) for detecting changes in ATOX1 level in primary neuronal cells at the cellular level (*n* = 3, one‐way ANOVA). h) RMSD change curves of ATOX1 during dynamic simulations. i) RMSD change curves of ligands relative to ATOX1. j) ATOX1 Rg value change curve. k) Trend of SASA of ATOX1 under the influence of different drugs with simulation time. l) Binding mode of ATOX1 and Ru‐EA. m) Typical plots of each group of in vitro experimental primary neurons after ATOX1 inhibition. n) Statistical graph of the total length of neuronal protrusions (mean ± SEM, n = 20, two‐way ANOVA). Scale bar = 50 µm; ^##^ *p* or **p < 0.01, ^###^ *p* or ***p < 0.001.

We used liquid chromatography‐mass spectrometry (LC‐MS) with proteomic analysis to analyze changes in the proteome of the spinal cord of mice in each group (**Figure** **6**a). Wayne and volcano plots of the differentially expressed proteins in the mouse spinal cord tissue are shown in Figure S7 (Supporting Information). Next, we identified the proteins associated with cellular oxidative stress using gene ontology (GO) enrichment analysis and analyzed their relative expression. Ru‐EA notably upregulated the ATOX1 protein, which showed considerable alteration. This protein is involved in cellular responses to oxidative stress among other functions (Figure 6b,c).

ATOX1 is a highly conserved water‐soluble protein that was first identified as a metal homeostatic factor in *Saccharomyces cerevisiae*. The protein is composed of two short α helices and four antiparallel β helices folded by classical iron redox folding (βαββαβαβ).^[^ ^50^ ^]^ ATOX1 delivers Cu ions to Cu‐dependent antioxidant enzymes such as SOD1. SOD1 is a key antioxidant enzyme that reduces the cellular damage caused by free radicals by converting superoxide anions into hydrogen peroxide and oxygen. Additionally, this process enables ATOX1 to protect cells from oxidative stress by regulating the Cu ion balance because excess Cu ions in cells generate harmful free radicals that lead to oxidative stress.^[^ ^20^, ^21^, ^51^, ^52^ ^]^

To validate the proteomic findings, western blotting was performed with the proteins extracted from primary neurons to determine whether Ru‐EA upregulated ATOX1 expression. The results showed that Ru‐EA significantly increased the expression of ATOX1 protein (Figure 6d,e). To enhance the reliability of the experimental results, we also extracted proteins from primary neurons for Western Blot analysis. As shown in Figure 6f,g, ATOX1 expression decreased in the post‐injury group compared with that of the control group, whereas it increased in the drug‐treated group with the Ru‐EA group showing a more pronounced effect. This indicates that Ru‐EA upregulates ATOX1 expression, and this effect was superior to that elicited by Ru‐PA. In summary, ATOX1 may be the primary protein target of Ru‐EA, and its expression may be upregulated. Moreover, atox1 functions as a novel transcription factor that, when activated by copper, undergoes nuclear translocation, binding to the novel cis element of the cyclin D1 promoter, and transactivation, thereby mediating effects of copper on cell proliferation. After spinal cord injury, tests for copper ions within the spinal cord revealed that, especially with the administration of Ru‐EA, the copper ion levels were closest to those of the control group and were higher than the injury group (Figure S8, Supporting Information).

### 2.10 Molecular Docking and Dynamics Simulations

The small molecule structural model used in this study is shown in Figure S9 (Supporting Information). ATOX1 and the two structurally similar but different complexes were subjected to docking compliance experiments. The optimal conformation was selected for 100‐ns dynamic simulations to investigate the differences in the binding of Ru‐PA and Ru‐EA to ATOX1. Figures S10 and S11 (Supporting Information) show snapshots of the structural changes during the simulation of the two molecules after binding to ATOX1. Ru‐PA and Ru‐EA bind to the protein through the bracketed portion as shown in Figure S9 (Supporting Information), whereas the PF^6^ portion did not bind to the protein during the kinetic simulation and remained free, suggesting that the bracketed portion of the two molecules primarily develops biological activity. Figures S10 and S11 (Supporting Information) show a difference between Ru‐PA and Ru‐EA in the tightness of the binding to the protein. Ru‐PA detached significantly from the protein during 90–100 ns of the simulation period, indicating that Ru‐PA molecules bind to the protein but the binding is not very stable. At 100 ns, the small molecule completely detached from the protein. However, Ru‐EA bound tightly to the protein through the entire duration of the simulation (100 ns), and the binding site remaining stable. This indicates that Ru‐EA could bind stably to ATOX1.

Furthermore, the root mean square deviation (RMSD) analysis of the complex system during the kinetic simulation is shown in Figure 6h, and it reflects the stability of the system. Figure 6i shows the change in the relative position of the small molecule with respect to the protein. This analysis shows that the stability of the Ru‐PA and Ru‐EA systems varied after the binding of the small molecules to the protein. The RMSD of Ru‐PA was >2 Å, whereas that of Ru‐EA was ≈2 Å, indicating that Ru‐EA binding is extremely stable, whereas that of Ru‐PA is unstable. The RMSD value of the relative protein of Ru‐PA increased slightly at ≈90–100 ns, indicating substantial detachment due to unstable binding. In contrast, the RMSD value of the relative protein of Ru‐EA showed no fluctuation, indicating more stable binding.

The radius of gyration (Rg) was used to measure the compactness of the atoms in the system in response to the stability of the binding of the small molecule to the protein. This provides an additional analysis of the effect of atom compactness. Higher Rg values indicate unstable binding, whereas lower Rg values indicate stable binding. As shown in Figure 6j, the Rg of the atoms in the Ru‐PA‐bound system was 11.12 Å, whereas the average Rg value of the atoms in the Ru‐EA‐bound system was 10.92 Å. This indicates that the Rg value of the system after binding of Ru‐PA was relatively larger than that after Ru‐EA binding, which reflects the better binding stability of Ru‐EA than that of Ru‐PA.

The solvent‐accessible surface areas (SASA) of the protein–small molecule complexes were analyzed. Å^2^ reflects the size of the exposed solvent area of the protein–small molecule binding system, which corresponds to the stability of the binding. A larger SASA indicates that more of the protein–small molecule complex is exposed to the solvent, suggesting decreased stability, whereas a smaller SASA indicates better structural stability and a larger embedded area. Figure 6k shows that after binding to protein, the average SASA of the complex is 4148.17 Å^2^ in the Ru‐PA system and 30 948.28 Å^2^ in the Ru‐EA system. This indicates the higher stability of the Ru‐EA binding complex than that of the Ru‐PA complex.

The degree of flexibility for each amino acid is shown in Figure S12 (Supporting Information). Root mean square fluctuation (RMSF), which measures the degree of freedom of movement of the atom and typifies the flexibility of the molecular structure, determines the degree of change of each atom in relation to its average location. The RMSF value is relatively stable with small fluctuations when the protein is stably bound to a small molecule, whereas it fluctuates more when the protein is unstably bound to a small molecule. Figure S12 (Supporting Information) shows the fluctuation in amino acids after the binding of both molecules to the protein. The data show that the RMSF of the protein fluctuates less after Ru‐EA binding, which may explain the more stable nature of the Ru‐EA binding.

Based on previous analyses, we found that the binding of small molecules to the protein leads to changes in protein structure. A difference was observed in the stability of the two molecules binding to the protein. The results indicate that slight changes occurred in the interactions within the protein; however, Ru‐PA and Ru‐EA did not show a very significant difference (Figure S13, Supporting Information).

We analyzed the effect of the two molecules on the differences in the protein secondary structure before and after the simulation (Figure S14, Supporting Information). We inferred From Figure S13 (Supporting Information), that the two molecules exerted a smaller effect on the internal amino acid interactions after binding to the protein, indicating that they exert a similarly smaller effect on the protein secondary structure, as shown in Figure S14 (Supporting Information). Although the secondary structural composition showed changes, the overall difference was not significant. However, compared with that of the empty protein system without molecular binding, the protein of the system with small molecules showed a certain degree of increase in β‐folding (E), which increased to 35.29%, and α‐helix content (H), which increased to 30.88%.

Subsequently, we analyzed the differences in the abilities of Ru‐PA and Ru‐EA to bind to proteins by first comparing the trends in the number of interactions with proteins during the simulations (Figure S15, Supporting Information). The analysis showed that four more classical interactions, namely, halogen bonding, hydrogen bonding, π‐π stacking, and ion‐π stacking interactions occurred between small molecules and proteins. The hydroxyl groups in the molecule acted as donors and acceptors of hydrogen bonds; the aromatic rings such as benzene rings acted as the basis for π‐π stacking and ion‐π stacking, whereas the F and Cl atoms acted as participants in halogen bonds. The data show that the Ru‐EA system possessed a higher number of protein–molecule interactions than the Ru‐PA system. The cumulative number of Ru‐PA–protein hydrogen bonds observed during the simulation was 394 with an average of 0.39, whereas the cumulative number of hydrogen bonds in Ru‐EA was 729 with an average of 0.72. This indicates a higher degree of Ru‐EA–protein interactions, which accounts for its higher stability than that of Ru‐PA during the simulation.

Furthermore, we compared the differences in their interactions when stably bound to the protein, and the Ru‐PA–protein binding pattern is shown in Figure S16 (Supporting Information). Ru‐PA forms a 3.0 Å hydrogen bonding interaction with the protein Glu13 amino acid. The other amino acids in close proximity are Ala16, Leu35, Asn32, Ile33, Glu30, Phe31, Ser20, and other amino acids that have formed van der Waals contacts and hydrophobic interactions. In the case of Ru‐EA–protein stable binding, the hydroxyl group and Glu13 form a hydrogen bonding interaction of 2.3 Å, which is closer than that of Ru‐PA (the closer the interaction, the stronger the effect). Additionally, Ru‐EA forms a π‐π stacking interaction with Phe31 at a distance of 5.1 Å. Chlorine atoms form a halogen bonding interaction with Ser20 at a distance of 4.0 Å with Asn32, Leu35, Ala16, and Ile33 in close proximity in the presence of van der Waals and hydrophobic interactions (Figure 6l).

Furthermore, the free energy of the binding of small molecules to ATOX1 during the simulation was calculated using the MM/GBSA method, as shown in Figure S17 (Supporting Information). The binding energies ΔG bind (ΔG total) of Ru‐PA and Ru‐EA to ATOX1 were −15.789 and −23.179 kcal mol^−1^, respectively. This shows that the binding energy of Ru‐EA was significantly larger than that of Ru‐PA because Ru‐PA had already detached from the protein after 90 ns, and the number of Ru‐PA interactions during the simulation period was less than that of Ru‐EA. Therefore, the value of the binding energy for the entire 100 ns period was much lower for Ru‐PA than for Ru‐EA.

### 2.11 Validation of Drug Interaction with ATOX1 Protein

DC_AC50 is a specific inhibitor of ATOX1 expression. We inhibited ATOX1 expression to determine if Ru‐EA requires ATOX1 to exert its effects.^[^ ^53^, ^54^ ^]^ DC_AC50 toxicity in HT22 cells and neurons was tested to determine the optimal working concentration. The results are presented in Figure S18 (Supporting Information). Then, DC_AC50 toxicity in primary neurons was tested (Figure S19, Supporting Information), and its inhibitory effect on ATOX1 in cells was verified (Figure S20, Supporting Information). A DCFH‐DA kit was used to detect the intracellular ROS levels in HT22 cells in each group. The results indicate that intracellular ROS level was elevated in the Inj+Ru‐EA+DC_AC50 group compared with that in the Inj+Ru‐EA group, suggesting that inhibition of ATOX1 expression reduces the Ru‐EA ROS scavenging effect (Figure S21, Supporting Information). Comparing the Inj+Ru‐EA+DC_AC50 and Inj group ROS levels, the former showed lower levels than that of the latter, indicating that Ru‐EA exerted ROS scavenging effect even after the inhibition of ATOX1 protein expression. This suggests that Ru‐EA may scavenge ROS both directly and by regulating ATOX1 expression. This is consistent with the results of the previous in vitro experiments, where Ru‐EA displayed antioxidant capacity in cell‐free systems. Possibly, the roles of DC_AC50 and Ru‐EA are not reciprocal in regulating ROS levels, and this warrants further investigation in future studies. Additionally, the experiment assessing the neuronal protective effects yielded similar results where despite the inhibition of ATOX1 expression, Ru‐EA continued to exert weakened neuronal protective effect (Figure 6m,n; Figure S22, Supporting Information). In summary, these results suggest that the mechanism underlying Ru‐EA‐exerted antioxidant effects involves the mitigation of ATOX1 expression; however, this may not be the only pathway as part of its antioxidant effects may be exerted by direct scavenging of ROS or through other currently unknown pathways. Further studies are required to clarify this.

## 3 Conclusion

This study has demonstrated that replacing η^6^‐Arene in bz‐PA with bz‐EA significantly improves the ROS scavenging ability and neuroprotective efficacy of ruthenium metal complexes. Additionally, Ru‐EA significantly stimulated ATOX1 production, resulting in an anti‐oxidative stress effect. In conclusion, Ru‐EA could protect neurons from SCI and provide a new therapeutic strategy for treating SCI.

## 4 Experimental Section

### Experimental Reagents and Animals

The brands, item numbers, or origins of the major experimental reagents and instruments used in this experiment were detailed in the Supporting Information.

Animals were purchased from the Laboratory Animal Center of Southern Medical Universit. The Institutional Animal Care and Use Committee of Jinan university approved all animal procedures (Approval No: IACUC‐20240603‐03). The mice were housed in appropriate cages with enough food and water, and their ideal temperature of 25 °C and 60% humidity was maintained. Throughout the whole experiment, female mice were utilized in order to provide voluntary urination following spinal cord damage. At the end of the experiment, the remaining mice were executed by cervical dislocation.

### Materials Synthesis and Characterization

Synthesis of the ligand: For one night, a mixture of methanol (5 mL), 2,6‐diisopropylaniline (5 mmol, 0.885 g), and a catalytic quantity of formic acid was mixed at room temperature. The mixture was named Pyridine‐2‐carboxaldehyde. After the solvent was completely evaporated using a rotary evaporator, a crude product was produced. This was then dried over anhydrous at room temperature and cleaned with five milliliters of water. The goods came as a powder that was yellow in color.

Synthesis of the Ru‐EA and Ru‐PA: In a dry round‐bottom flask with a nitrogen atmosphere and stirrer, the ligand 2,6‐diisopropyl‐N‐(6‐fluoroquinolin‐2‐ylmethylene)aniline (0.10 mmol) and metal dimer [(η^6^‐arene)RuCl_2_]2 (0.05 mmol) were dissolved in methanol. Room temperature NH_4_PF_6_ (0.2 mmol) was added after a 12‐hour period of continuous stirring.

The reaction mixture was stirred for 6 h at RT. The product was dissolved and filtered through a funnel, then recrystallized by slow diffusion of n‐hexane to give the corresponding complexes after complete conversion. Finally methanol was removed under reduced pressure (Ru‐EA and Ru‐PA, see Supporting Information for details).

### Free Radical Scavenging Assays

The antioxidant capacity of the materials were evaluated using ABTS [2,2'‐azinobis‐(3‐ethylbenzothiazoline‐6‐sulfonate)] and DPPH [2,2‐Diphenyl‐1‐picrylhydrazyl] free radical assays. The ABTS stock solution (in PBS) and manganese dioxide solution were mixed to produce ABTS radicals using the method in the previous study.^[^ ^55^ ^]^ Prepare DPPH as a highly concentrated storage solution (in ethanol) according to the instructions for use, and dilute it for use. Dilute the ABTS or DPPH storage solution to the appropriate concentration according to the instructions, and then mix it with different concentrations of drugs.

### Cultivation of Primary Neurons

P0 Sprague Dawley rats were beheaded as a kind of sacrifice, and the hippocampi were removed from the brains. The hippocampal tissue was immersed in 0.125 percent trypsin and immersed for 20 min at 37 °C in a water bath. Trypsin digestion was stopped by adding 10% fetal bovine serum to DMEM/F12. The cells were grown at a density of 1 × 10^4^ cells cm^2^. After 6 h, the medium was swapped out with neurobasal‐A medium supplemented with two percent B‐27. The cells were cultivated with 5% CO^2^ at 37 °C.

### Cell Toxicity Assay

The CCK‐8 was used to test the toxicity of the drugs to the cells in order to determine the concentration to be used. First, after the cells were fully appended to the wall in the 96‐well plate species, they were incubated with cell culture medium containing different concentrations of drugs for as long as required for the experiment. At the end of incubation, the original medium was discarded, the pre‐prepared CCK‐8 reagent was added, and the 96‐well plate was placed in a cell incubator. Absorbance was measured after 40 min and cell viability was calculated from it.

### Detection of Reactive Oxygen Species and Superoxide Anion in Cell

The HT22 cells were incubated in a complete medium containing glutamate to induce injury and mimic the desired state of oxidative stress. Subsequently, the cells in the oxidative stress state were treated with Ru‐EA and Ru‐PA. Finally, the antioxidant effects exerted by Ru‐EA and Ru‐PA on the HT22 cells were assessed by measuring the ROS levels. We used DCFH‐DA and DHE kits to detect total intracellular ROS and superoxide anion, respectively. Cells were seeded in 24‐well plates, and after the cells were fully appressed to the wall, the cells were incubated for 12 h using glutamate‐containing medium (120 mM) in order to induce an oxidative stress state in the cells. After that, the cells were immersed again with the drug for 12 h. Finally, the original medium was discarded and the DCHF‐DA or DHE reagent was prepared according to the instructions of the kit and added to the 24‐well plate for 25 min. After that, the cells were incubated in 4% paraformaldehyde for 45 min. Then, the cells were fixed with DAPI‐containing sealing agent.

### Neuroprotective Effects in Vitro

Primary neurons were cultured for 48 h at 37 °C in a 5% CO^2^ environment. They were first treated with glutamate (120 µM) for 12 h to induce oxidative stress injury. Next, the original culture medium was discarded and the neurons were continued to be cultured using medium containing different concentrations of drugs for 12 h. The neurons were incubated with primary antibody against β3‐Tubulin (1:1000) and subsequently with secondary antibody (1:2000) at RT for 2 h. Coverslips were attached with a Fluorescent‐Gel II containing DAPI and imaged by confocal microscopy. The length and number of primary and secondary protrusions of each grouped neuron were recorded using ImageJ software.

### Animal Models of SCI

Mice were grouped according to experimental requirements. For SCI modelling in the experimental group, mice were first anaesthetised with 3% tribromoethanol intraperitoneally (200 µL/20 g). After successful anaesthesia, the hair in the surgical area was removed, followed by sequential incision of the skin, muscle and vertebral plate to expose the spinal cord tissue (T8‐10). The parameters of the spinal cord impactor were adjusted, i.e., impaction depth of 1 mm and impaction time of 0.5 s, and the spinal cord of the T8‐10 segments was implanted. After the impingement, a haematoma could be observed at the impinged site of the spinal cord, and the hind limbs of the mice were paralyzed, after which the wound was closed layer by layer. The sham group only exposed the spinal cord without impingement injury to it. The postoperative treatment group was treated with the appropriate drug (312.5 µM, 200 µL) once a day for 7 days.^[^ ^56^ ^]^

### Detection of Oxidative Stress Levels in Vivo

On the day 3, groups of mice were injected with a chemiluminescent probe called L‐012 (75 mg k^−1^g). mice were anesthetized after 15 min. Afterward, each group of mice was placed in a small animal imaging system and the level of ROS in the spinal cord region of the mice was observed.

### Behavioral Experiments with Mice

Behavioral experiments were implemented over a 60‐day postoperative period to assess motor recovery of both hindlimbs in mice. Specific experiments included Basso Mice Scale (BMS) scoring, footprinting experiments, inclined plate experiments, and Deeplabcut‐involved mouse bilateral hindlimb movement trajectory analysis.

Scoring was performed every 3 days using the BMS scoring to determine the hindlimb motor recovery of the mice. In the footprint experiment, we applied blue printing oil to the bilateral soles of the mice and allowed them to crawl on a paper, after which the footprints on the paper were scanned and analyzed. In the inclined plate experiment, mice were placed on a flat plate that could be gradually tilted at increasing angles, and the angle of the plate to the horizontal was recorded as the mice slid off the plate, after which the angle at which the mice slid off the plate was grouped in each group.

### Detection of Joint Mobility in SCI Mice

DeepLabCut (version 2.3.7) was used to clip and crop the collected videos of mouse hindlimb activity to extract key frames. The sacroiliac, hip, knee, ankle, and toes of mice were manually identified and labeled. The dataset was constructed using mobnet to train the model, assessing the network loss rate below 0.001% then into the analysis of the complete video, checking the accuracy of the labeled positions, and analyzing the average crawling speed, average joint height and joint mobility of the mice in each group.

### Electrophysiological Experiments in Mice

Electrophysiological experiments were used to assess the recovery of motor evoked potentials and spinal electrical signal conduction after SCI in mice. To assess motor evoked potentials, mice were anaesthetized and a window of ≈5 mm in diameter was opened on both sides of the skull fontanelle using a milling drill, thereby exposing the cerebral cortex bilaterally. Recording electrodes were positioned in the right hindlimb's gastrocnemius muscle after stimulating electrodes were first inserted 500 µm deep into the left cerebral cortex's motor region. The Biosignal Acquisition System was then linked to them, and the necessary settings, were made in order to capture the motor evoked potentials. The method of acquiring the right motor evoked potentials was the same as that for the right side.

The methodology employed to evaluate the conduction of electrical signals in the spinal cord was akin to that of the preceding study. The mice's backs were slashed open, exposing the spinal cord, and the damaged spinal cord segments were identified. The spinal cord tissue proximal to the injured segments was stimulated with stimulating electrodes, and the potentials were recorded proximal to the injured segments, 2 mm distal to the injured segments and 5 mm distal to the injured segments, respectively, and the spinal cord electrical transmission was evaluated according to the amplitude of potentials.

### Tissue Sections of Mice

At day 60, the mice were necropsied, and the spinal cord was then sectioned. Some slices were stained with H&E and LFB, and the results were observed on video under a light microscope. Some sections were incubated with the corresponding primary antibodies, such as anti‐Iba‐1 (1:200), anti‐CD68 (1:400), anti‐iNOS (1:200), anti‐NFH (1:200), and anti‐GFAP (1:400), as well as the corresponding secondary antibodies (Alexa Fluor 555 and 488 [1:1000]), and then washed off the antibodies and sealed with DAPI‐containing sealing agent, and finally photographed using a Confocal microscopy was used to take pictures (details of the antibodies were given in the Supporting Information).

The spleen, kidney, heart, lung, liver, and brain tissues were obtained, they were paraffin embedded, sectioned as well as stained with H&E and finally the slides were scanned and observed.

### Proteomics Analysis

On day three, the spinal cord was extracted within a 1 cm radius of each mouse. Subsequently, the tissue was soaked in saline to remove surface tissue. The spinal cord tissue was then lysed at 4 °C for half an hour using a lysis solution. The tissue was then centrifuged at 4 °C for 20 min and the supernatant is collected. The next steps were proteolysis, digestion and desalting to convert proteins to peptides and remove impurities. Following LC‐MS analysis of the materials, DIA was used to analyze the results. R was used for bioinformatics analysis to determine which proteins the medication mostly impacts.

### Western Blotting

First, the corresponding cells or tissues were lysed at 4 °C using lysis buffer, after which the supernatant was centrifuged and taken. Then 5×Loading buffer was added to the supernatant and boiled at 95 °C for 5–10 min in a dry thermometer to obtain the protein samples.

After that, a 7.5% SDS‐PAGE gel was prepared according to the instructions of the one‐step gel dispensing kit, protein samples were added to the corresponding wells according to the grouping, and electrophoresis was performed by connecting the power supply and adjusting the appropriate voltage. Following electrophoresis, the samples were moved onto a PVDF membrane through electrotransfer. Subsequently, the PVDF membrane underwent blocking at room temperature for 15 minutes using a protein‐free fast blocking buffer. Next, the membranes were treated with primary antibodies (Beta‐Tubulin [1:5000]) at 4 °C for 12 hours, then rinsed twice with TBST and exposed to the appropriate secondary antibody (1:5000) at RT for 2 h. Finally, visualization was achieved by exposing the membranes to BeyoECL Plus (details of the antibodies are given in the Supporting Information).

### Molecular Docking

The ATOX1 protein was structurally modelled using Alphafold2,^[^ ^57^ ^]^ retaining regions of high confidence. Then, molecular docking experiments were implemented using AutoDock 4.2, and the optimal binding site was predicted using SiteMap software, and the predicted binding site was set as the docking center and the corresponding parameters were set. Afterward, the conformations were sampled and scored using genetic algorithms, and the optimal conformations were selected based on the docking scores of the conformations sorted.

### Molecular Dynamics Simulation

In order to investigate the binding effect of these two molecules on the ATOX1 protein and to compare the differences between them, we performed molecular dynamics simulations using Gromacs 5.1.5. The system was set up in a closed environment and the appropriate parameters were set. Post NVT and NPT equilibrium, kinetic simulations spanning 100 ns were performed, with a time step of 2 fs for system simulations. Covalent bond lengths were constrained using a linear constraint solving algorithm, while long‐range electrostatic interactions were managed via the PME method. Once all simulations concluded, the gmx module facilitated the computation of Rg, hydrophobic contact interactions, in addition to RMSD and RMSF analyses.

### Calculate the Binding Free Energy

The ΔG bind of protein molecules and ligands was calculated in this study using Molecular mechanics with a generalized Born and surface area solvation approach:

(1) ΔGbind=ΔH−TΔS≈ΔGsolv+ΔGGAS−TΔS

(2) ΔGGAS=ΔEint+ΔEvdw+ΔEele

(3) ΔGsolv=ΔEsurf+ΔEGB

### Statistical Analysis

Statistical analyses such as normal distribution and chi‐square, parametric and non‐parametric tests were performed using SPSS statistics 26.0 and the results were imported into Graphpad prism 9.0 for visualization. To calculate the fluorescence intensity as well as the length and quantity of neuronal protrusions, Image Pro was utilized. All data were presented as mean ± standard deviation unless specified otherwise. P < 0.05 was regarded as statistically significant.

## Conflict of Interest

The authors declare no conflict of interest.

## Supporting information



Supporting Information

## Data Availability

The data that support the findings of this study are available from the corresponding author upon reasonable request.
