# Supplementary material for: Metal Ruthenium Complexes Treat Spinal Cord Injury By Alleviating Oxidative Stress Through Interaction With Antioxidant 1 Copper Chaperone Protein
Source: Adv Sci (Weinh). 2024 Oct 16;11(45):2407225. doi: 10.1002/advs.202407225 (PMC11615763; doi:10.1002/advs.202407225)
Supplement: Supplementary file 1 — Supporting Information [file ADVS-11-2407225-s001.pdf]

ADVANCED SCIENCE
Open Access

Supporting Information

for *Adv. Sci.*, DOI 10.1002/advs.202407225

Metal Ruthenium Complexes Treat Spinal Cord Injury By Alleviating Oxidative Stress Through Interaction With Antioxidant 1 Copper Chaperone Protein

Juanjuan Li, Cheng Peng, Caiqiang Huang, Li Wan, Ke Wang, Ping Wu, Tianjun Chen, Guodong Sun, Rui Guo*, Hongsheng Lin* and Zhisheng Ji**

Supplementary materials

Main experimental reagents details:

2,2 '-azinobis-(3-ethylbenzothiazoline-6-sulfonate) (ABTS, MCE, cat. no. #HY-15902)

2,2-Diphenyl-1-picrylhydrazyl (DPPH, MCE, cat. no. #HY-112053)

Dulbecco's modified Eagle medium (DMEM) medium (Gibco, cat. no. #C11995500BT)

Fetal bovine serum (Gibco, cat. no. #10270-106)

DMEM/F12 (Gibco, cat. no. #11995065)

Poly-D-lysine (Sigma, cat. no. #P6407)

Neurobasal-A media (Gibco, cat. no. #10888022)

B-27 supplements (Gibco, cat. no. #17504044)

Lysis buffer (Beyotime, cat. no.#P0013)

Polyvinylidene fluoride membrane (Millipore, cat. no. #IPVH00010)

Protein free rapid blocking buffer (EpiZyme, cat. no. #PS108P)

Tween-20 (Meilunbio, cat.no. #MB2483)

BeyoECL Plus (Beyotime, cat. no. #P0018S)

Paraformaldehyde (Meilunbio, cat. no. #MA0192)

Dichlorodihydrofluorescein diacetate kit (DCFH-DA, Beyotime, cat. no. #S0033S)

Dihydroethidium kit (DHE, Beyotime, cat. no. #S0063)

Glutamic acid (Sigma, cat. no. #49621)

0.125% trypsin (Gibco, cat. no. #15400054)

Cell counting kit-8 (CCK-8, Beyotime, cat. no. #C0038)

Fluoro-Gel ll with DAPI (Electron Microscopy Science, cat. no. #17985-50)

L-012 (Wako, cat. no. #120-04891)

DC_AC50 (MCE, cat. no. #HY-107636)

NFH antibodies (Abcam,cat. no. #ab207176)

GFAP antibodies (Abcam,cat. no. #ab7260)

Iba-1 antibodies (Abcam, cat. no. #ab5076)

CD68 antibodies (Abcam,cat. no. #ab283654)

iNOS antibodies (Abcam,cat. no. #ab178945)

Beta 3-Tubulin antibodies (Abcam, cat. no. #ab78078)

Beta-Tubulin antibodies (Abcam, cat. no. #ab6046)

HRP Goat Anti-mouse IgG (H + L) (Abclonal Biotechnology, cat. no. #AS003)

HRP GoatAnti-rabbit IgG (H + L) (Abclonal Biotechnology cat. no. #AS014)

Alexa Fluor 555 (Thermo Fisher Scientific, cat. no. #A-21428)

Alexa Fluor 488 (Thermo Fisher Scientific, cat. no. #A-10680)

ATOX1 antibodies (Abcam, cat. no. #ab154179)

Synthesis of Ru-PA and Ru-EA:

2,6-diisopropyl-N-(6-fluoroquinolin-2-ylmethylene)aniline. ^{1}H NMR (500 MHz, $CDCl_3$) δ 8.51 – 8.41 (m, 2H), 8.29 – 8.18 (m, 2H), 7.54 (dt, J = 11.6, 5.7 Hz, 2H), 7.22 – 7.11 (m, 3H), 2.99 (dt, J = 13.7, 6.9 Hz, 2H), 1.28 (d, J = 6.8 Hz, 1H), 1.19 (d, J = 6.9 Hz, 11H).

[(η^6-bz-PA)Ru(L1)Cl]PF_6 (Ru-PA): ^{1}H NMR (500 MHz, DMSO) δ 9.24 (s, 1H), 8.92 (d, J = 8.3 Hz, 1H), 8.79 (dd, J = 9.6, 5.0 Hz, 1H), 8.38 (d, J = 8.3 Hz, 1H), 8.21 (dd, J = 8.7, 2.9 Hz, 1H), 8.10 – 8.03 (m, 1H), 7.63 – 7.51 (m, 2H), 7.51 – 7.44 (m, 1H), 6.49 – 6.36 (m, 2H), 5.74 (dd, J = 11.5, 5.9 Hz, 1H), 5.35 – 5.24 (m, 2H), 4.62 (t, J = 5.0 Hz, 1H), 3.82 (dt, J = 16.6, 8.2 Hz, 1H), 3.49 – 3.40 (m, 2H), 2.77 – 2.70 (m, 1H), 2.43 (dd, J = 13.3, 7.6 Hz, 2H), 1.83 – 1.71 (m, 2H), 1.40 (d, J = 6.7 Hz, 3H), 1.28 (dd, J = 29.0, 6.7 Hz, 6H), 0.88 (d, J = 6.7 Hz, 3H). Anal. Calcd. For [(η^6-bz-PA)Ru(L1)Cl]PF_6 (752.11): C, 49.51; H, 4.69;N, 3.72; Found: C, 49.53; H, 4.67;N, 3.70. MS: m/z 573.18. [(η^6-bz-PA)Ru(L1)+H]$^+$.

[(η^6-bz-EA)Ru(L1)Cl]PF_6 (Ru-EA): ^{1}H NMR (500 MHz, DMSO) δ 9.25 (s, 1H), 8.93 (d, J = 8.3 Hz, 1H), 8.80 (dd, J = 9.7, 4.9 Hz, 1H), 8.38 (d, J = 8.3 Hz, 1H), 8.24 – 8.19 (m, 1H), 8.10 – 8.05 (m, 1H), 7.57 (dd, J = 7.0, 3.9 Hz, 2H), 7.48 (dd, J = 6.0, 3.2 Hz, 1H), 6.47 – 6.38 (m, 2H), 5.78 – 5.76 (m, 1H), 5.36 (t, J = 5.8 Hz, 1H), 5.29 (d, J = 6.0 Hz, 1H), 4.89 (t, J = 5.0 Hz, 1H), 3.84 – 3.78 (m, 1H), 3.72 (dd, J = 11.4,

5.7 Hz, 2H), 2.78 (dd, J = 11.8, 6.3 Hz, 1H), 2.69 – 2.58 (m, 2H), 1.40 (d, J = 6.7 Hz, 3H), 1.28 (dd, J = 27.5, 6.7 Hz, 6H), 0.87 (d, J = 6.7 Hz, 3H). Anal. Calcd. For [(η^6-bz-EA)Ru(L1)Cl]PF_6 (738.10): C, 48.82; H, 4.51;N, 3.80; Found: C, 48.80; H, 4.54;N, 3.82. MS: m/z 559.16. [(η^6-bz-EA)Ru(L1)+H]$^+$.

Supplementary figures:

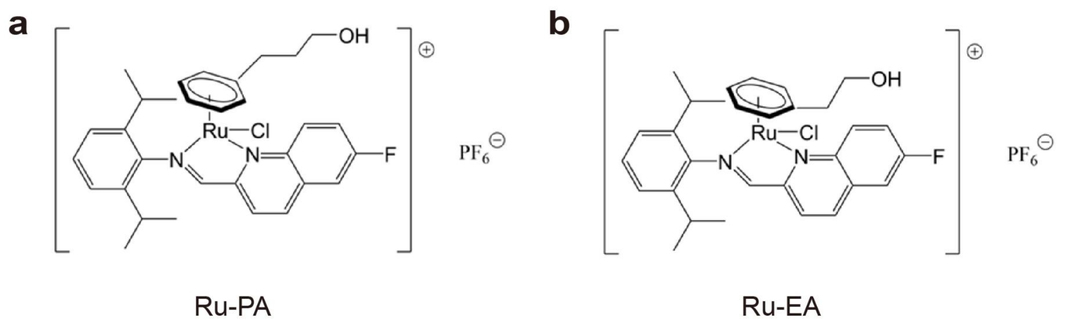

Fig.S1 Structural formulae of two metallic ruthenium complexes.

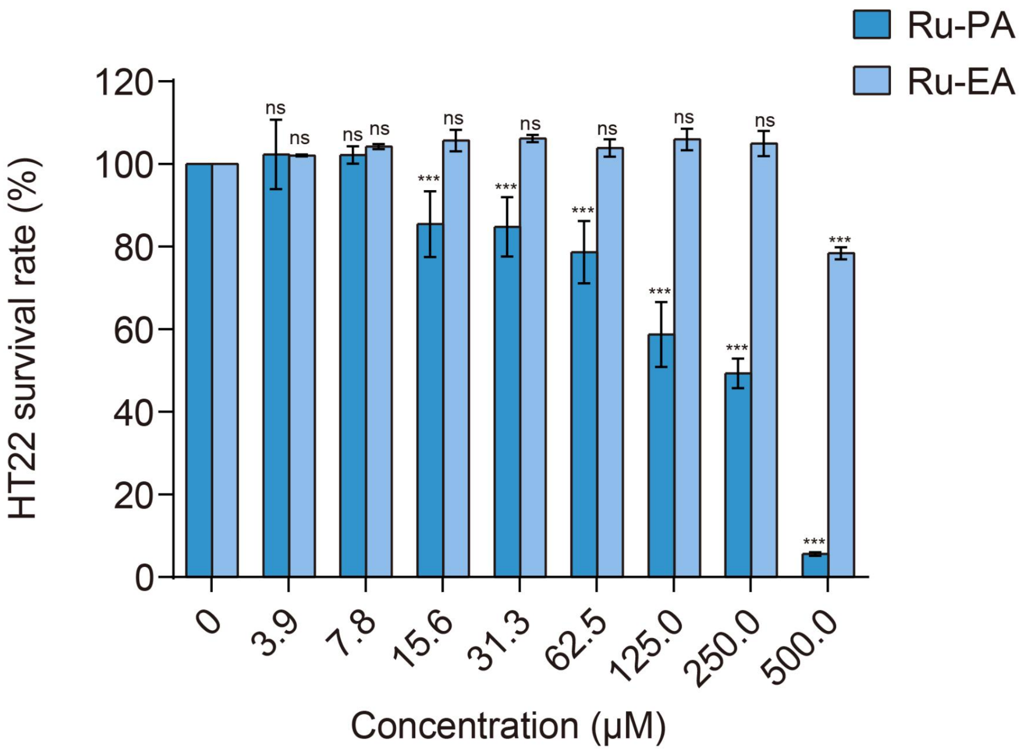

Fig.S2 Toxicity assay of Ru-EA and Ru-PA on HT22 cells. CCK-8 kit assay showing the effect of different concentrations of the two drugs on the viability of

HT22 cells (n = 3). ***$P < 0.001$ vs 0 μM. (n represents the number of samples in each group).

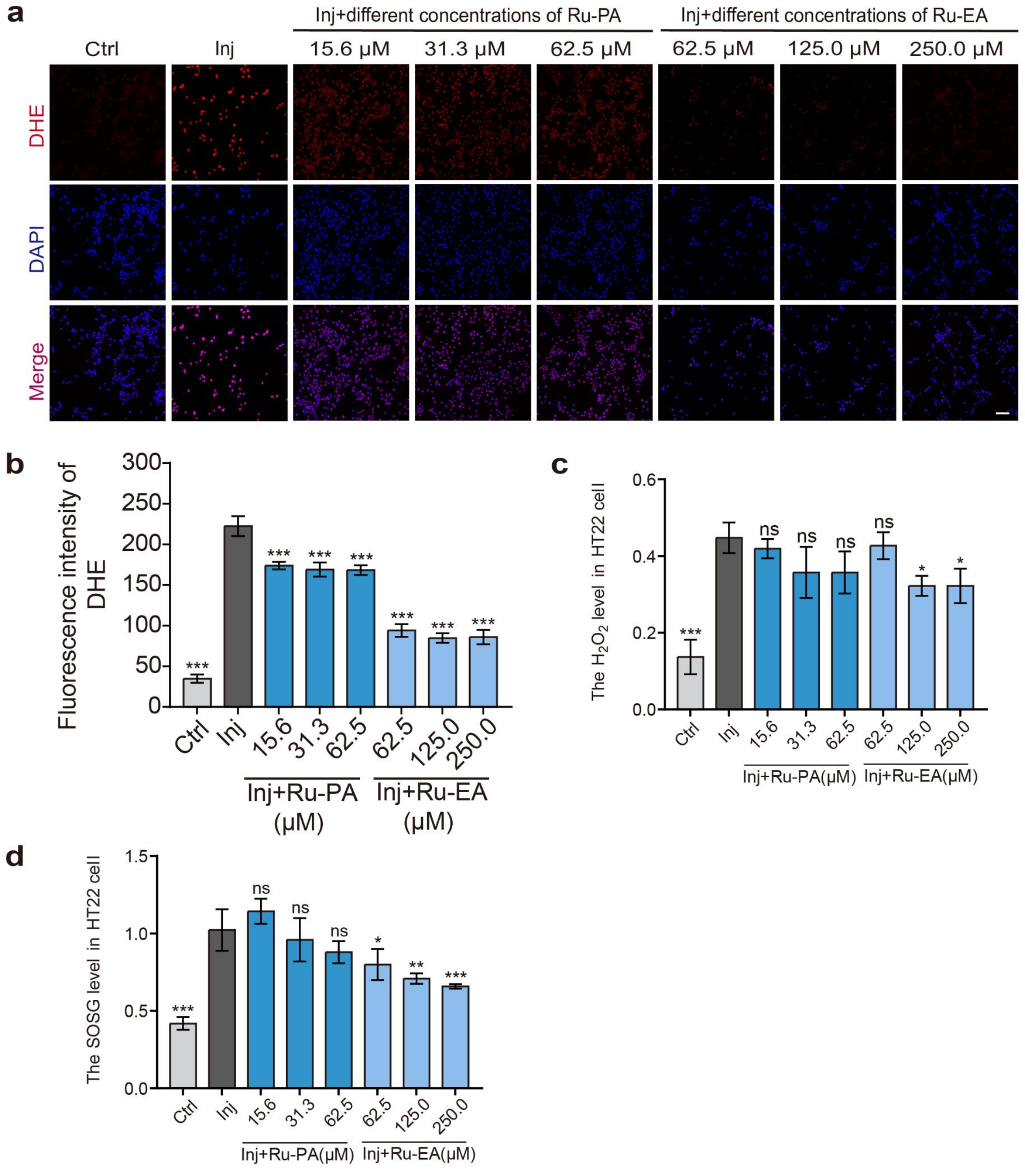

Fig.S3 Representative plots of the regulatory effects of Ru-EA and Ru-PA on intracellular superoxide anion levels. (a) Levels of superoxide anion after the action of different concentrations of Ru-EA and Ru-PA on HT22 cells as determined by DHE, scale bar = 100 μm. (b) Statistical plots of fluorescence intensity after treatment of HT22 cells with different concentrations of Ru-EA and Ru-PA (n = 5). ***$P < 0.001$ vs inj (inj = injury group). (c) Relative levels of $^{1}O_2$ in HT22 cells after treatment with

different concentrations of Ru-EA and Ru-PA test by SOSG (Singlet Oxygen Sensor Green reagent), n=3, ***P<0.001 vs injury group. (d) Relative levels of H_2O_2 in HT22 cells after treatment with different concentrations of Ru-EA and Ru-PA, n=3, ***P<0.001 vs injury group.

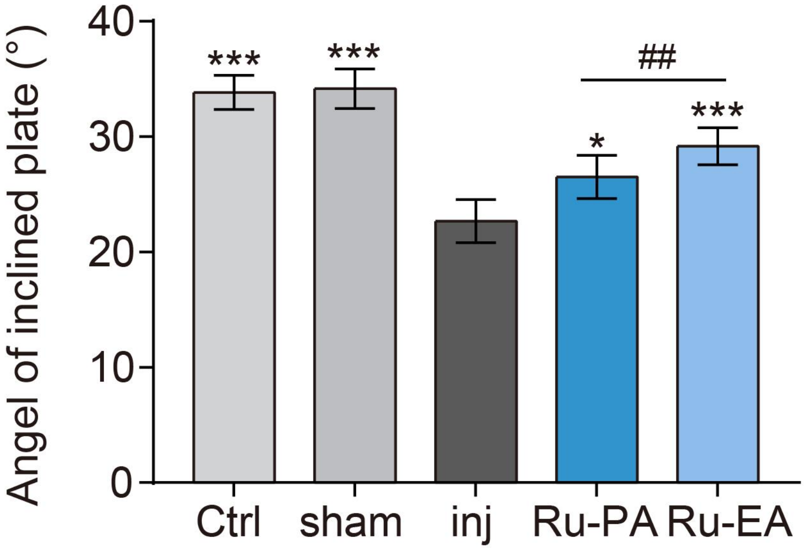

Fig.S4 Mouse inclined plate experiment. Statistical plot of the maximum angle at which each group of mice could hold on to the plate at progressively increasing angles of inclination without dropping it (n=6). ***P < 0.001 vs inj; $^{\#\#\#}P$ < 0.001 vs Ru-PA.

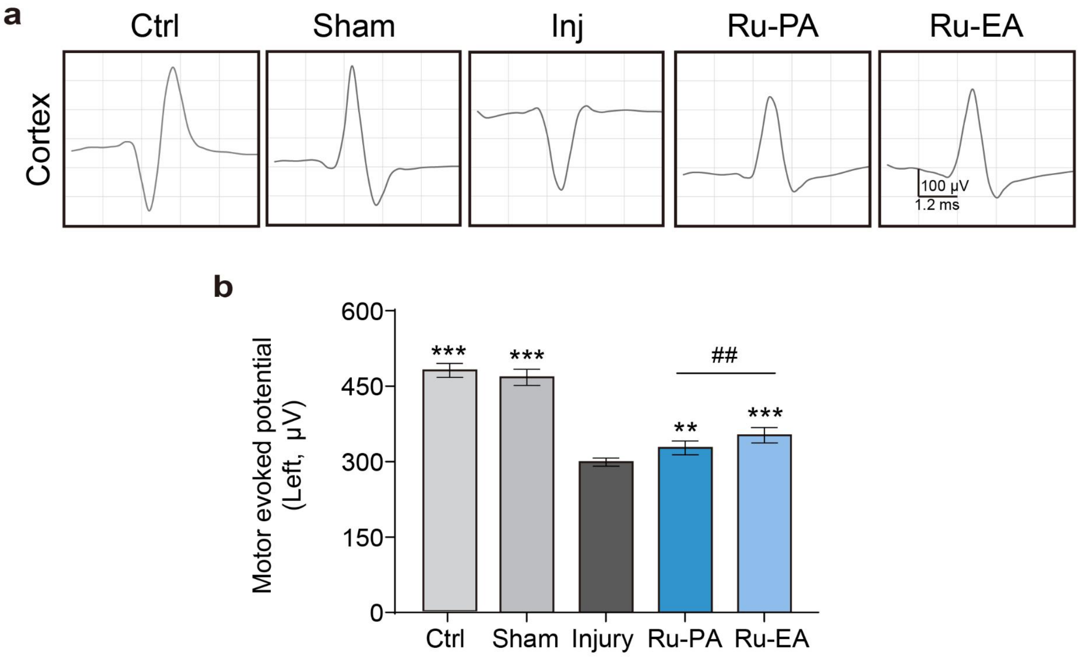

Fig.S5 Motor evoked potentials in the left cortex of mice. (a) Representative images of electrophysiological waveforms in the left cortex of mice. (b) Mouse left cortical motor evoked potentials (mean ± SEM, n = 6). **$P < 0.01$, ***$P < 0.001$ vs inj; $^{\#\#}P < 0.01$ vs Ru-PA.

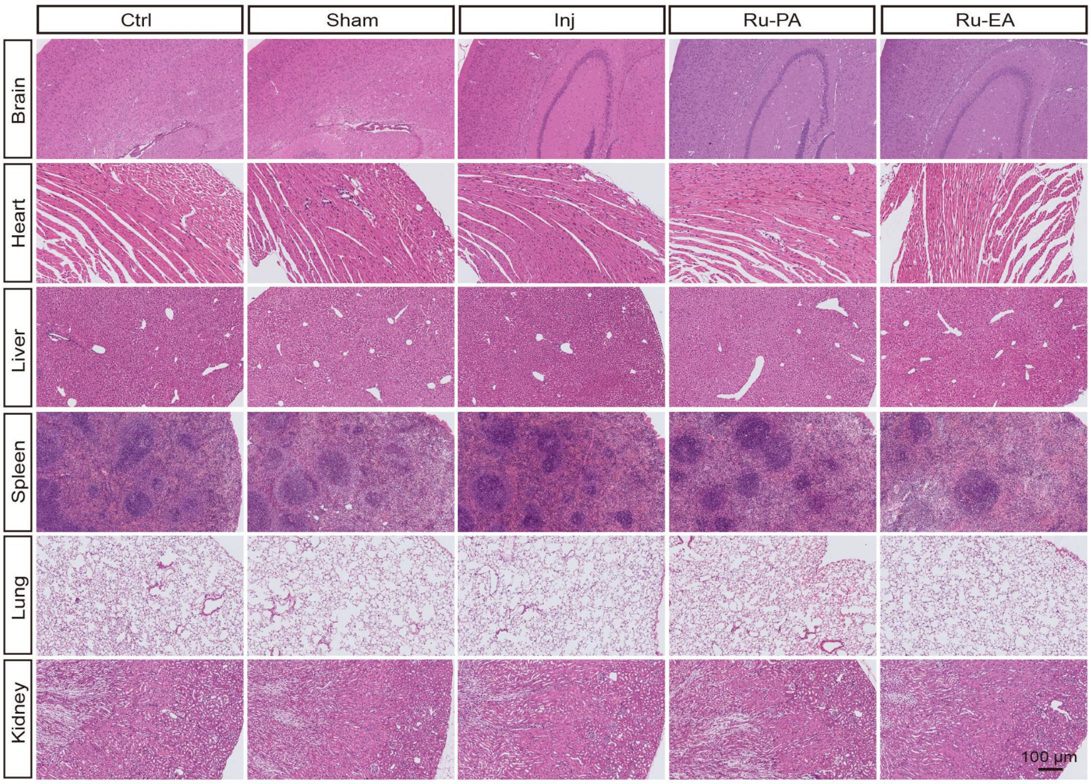

Fig.S6 Biocompatibility of drugs on mouse brain, heart, liver, spleen, lungs and kidneys. H&E stained sections of brain, heart, liver, spleen, lungs and kidneys of each group of mice at day 60, scale bar = 100 μm.

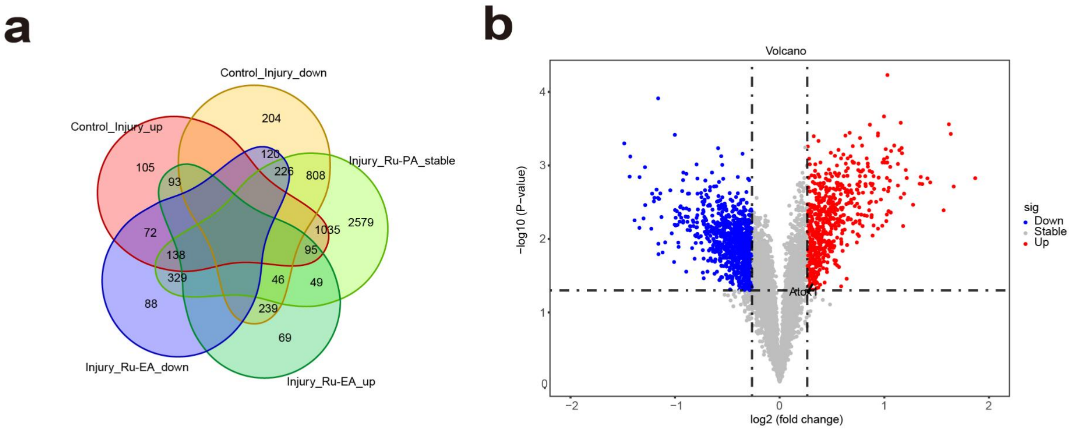

Fig.S7 Proteomics analysis. (a) Wayne diagrams for proteomics analysis. (b) Volcano plots of the expression of proteins interacting with drugs (p-value < 0.05 & foldchange > 1.5).

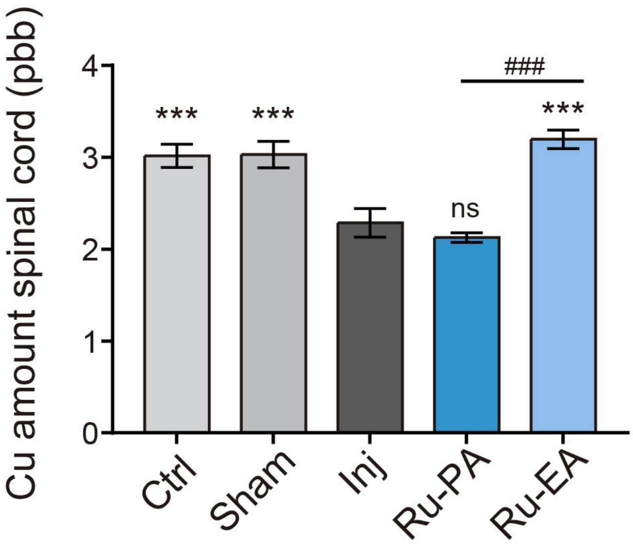

Fig.S8. Cu amount in spinal cord of mice after treatment with Ru-EA and Ru-PA, n=3, ***P<0.001 vs inj group test by ICP-MS.

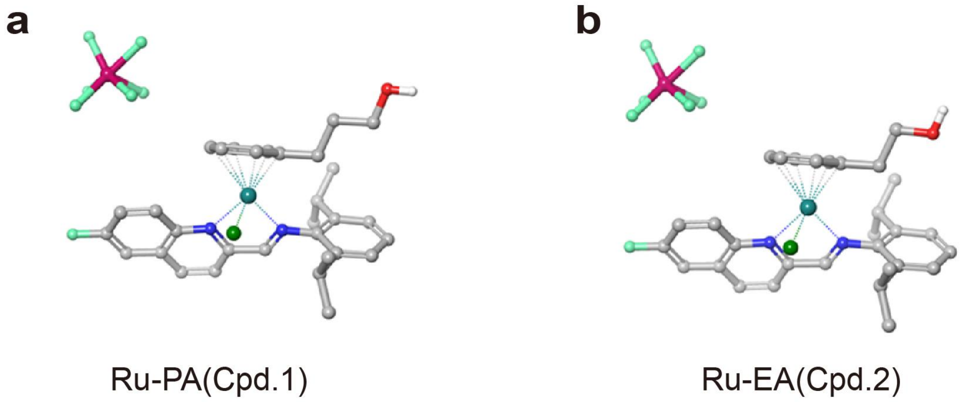

Fig.S9 Small molecule structural models used in this study. (a) Structural model of Ru-PA. (b) Structural model of Ru-EA.

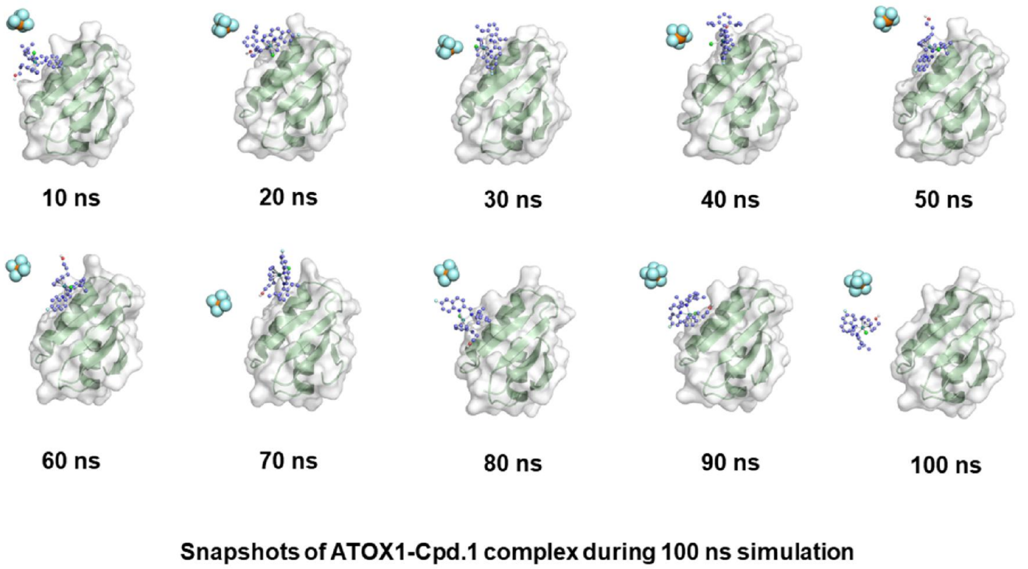

Fig.S10 Snapshots of structural changes during 100ns simulation of ATOX1-Ru-PA complex kinetics. The proteins are shown using the light green Cartoon+transparent Surface display mode, Cpd1 is shown using the blue Sphere, and the PF6 of the complex in Ru-PA is shown with cyan blobs.

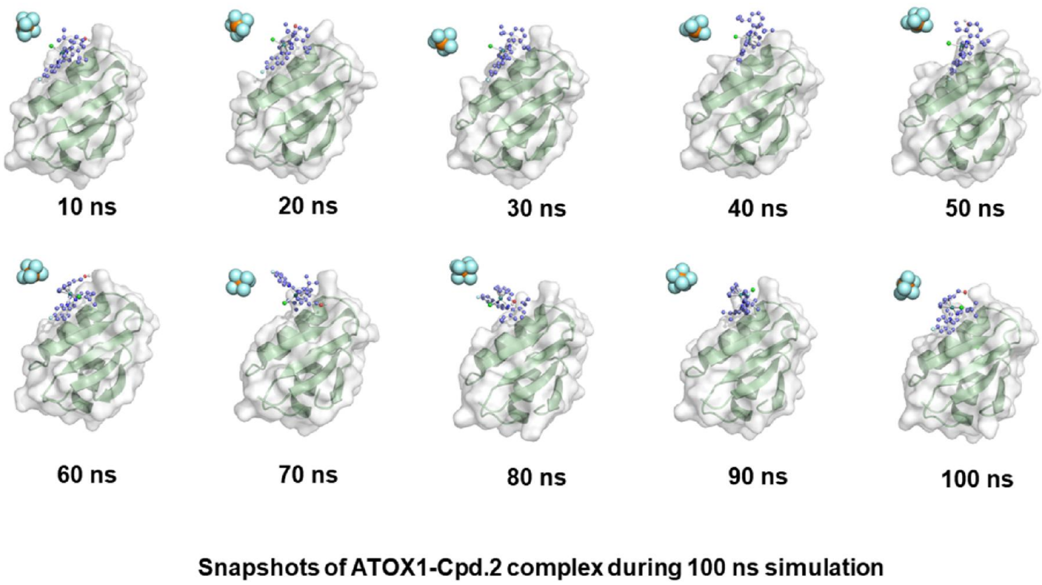

Fig.S11 Snapshots of structural changes during 100ns simulations of ATOX1-Ru-EA complex dynamics.

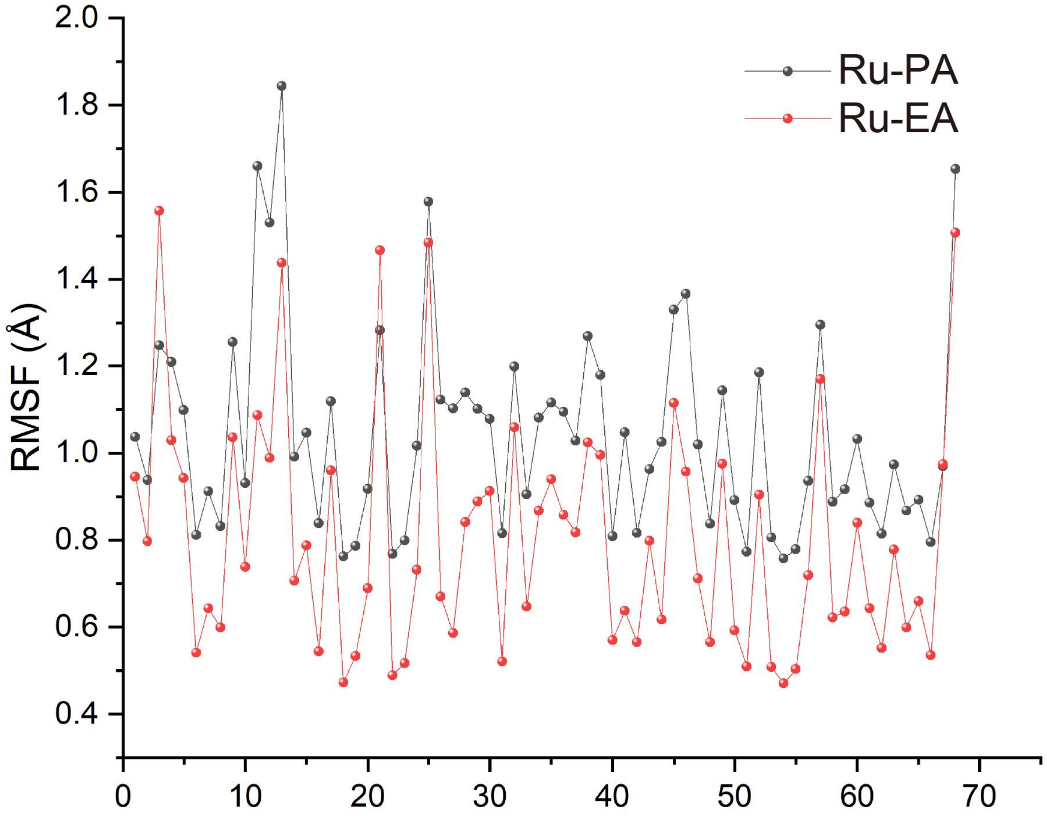

Fig.S12 Comparison of protein amino acid residue stability RMSF after binding Ru-PA and Ru-EA.

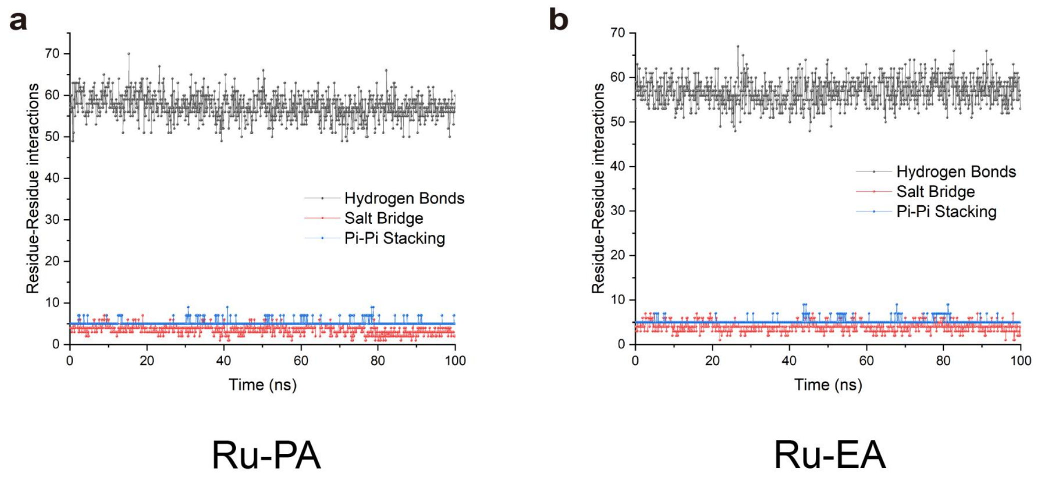

Fig.S13 Effect of two drugs on amino acid interactions within the ATOX1 protein.
(a) Ru-PA. (b) Ru-EA.

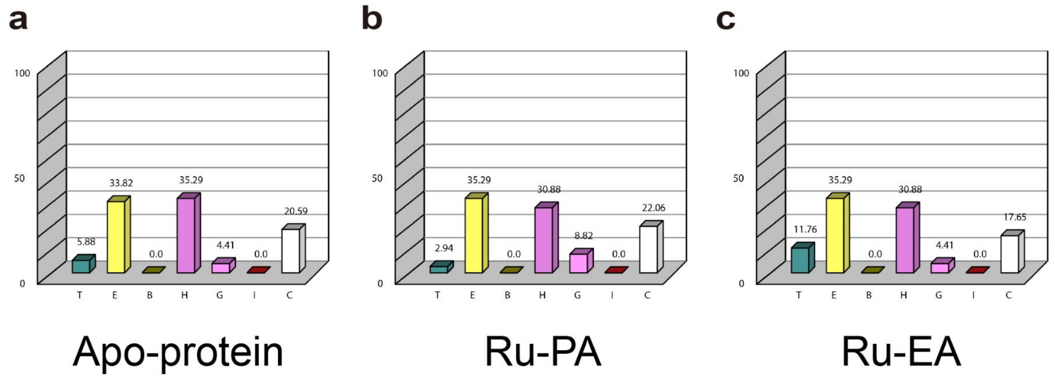

Fig.S14 Comparison of the structural changes of the complexes before and after kinetic simulation. Apo protein (a) is the secondary structure of the protein before the simulation, and Ru-PA (b) and Ru-EA (c) are the secondary structures of the proteins in the two systems after the simulation of 100 ns. T: β-folding corner, E: β-folding lamellae, B: β-folding bridges; H: α-helix; G: 3_{10}-helix; I: π-helix; C: random curling.

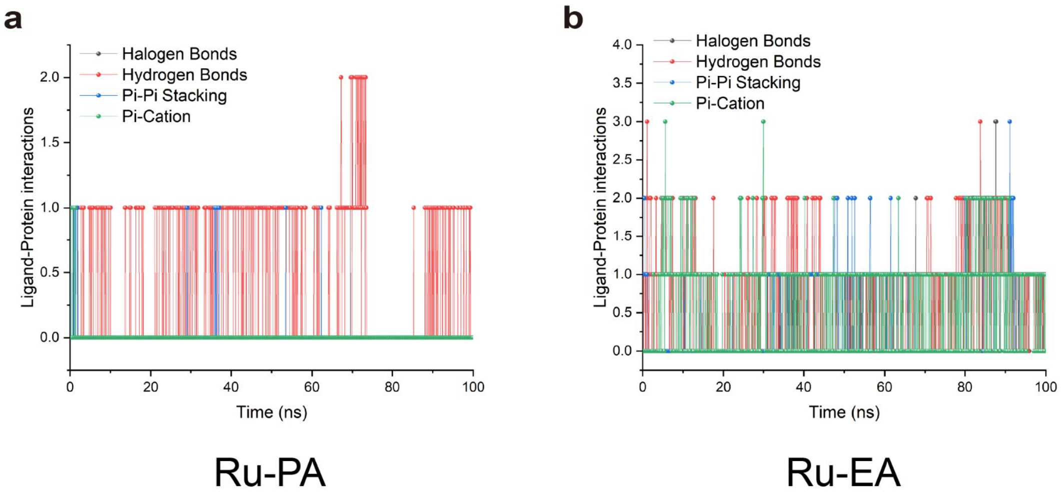

Fig.S15 Comparison of differences in the number of drugs and proteins wanting to work well. (a) Ru-PA. (b) Ru-EA.

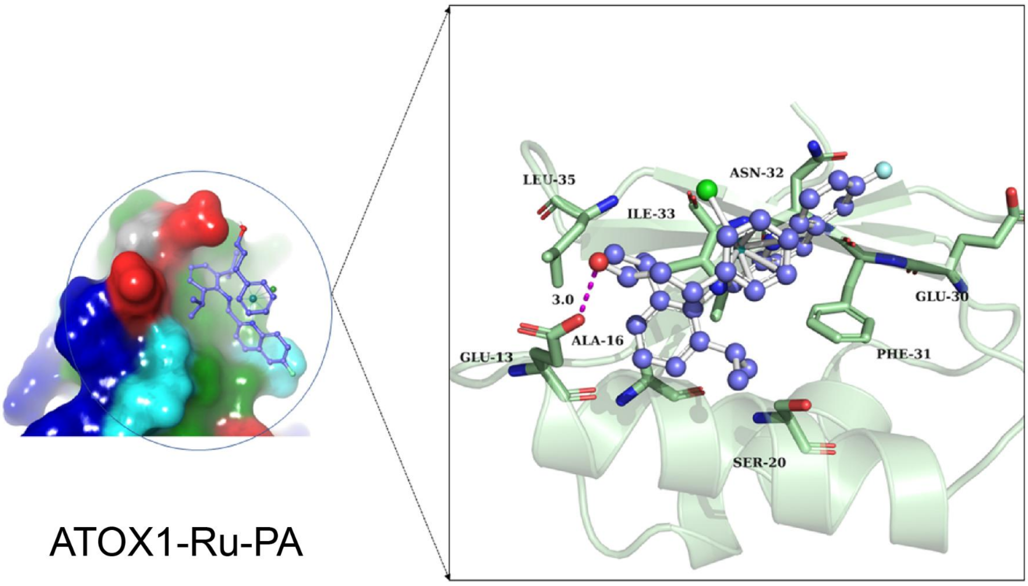

Fig.S16 Binding mode of ATOX1 protein and Ru-PA.

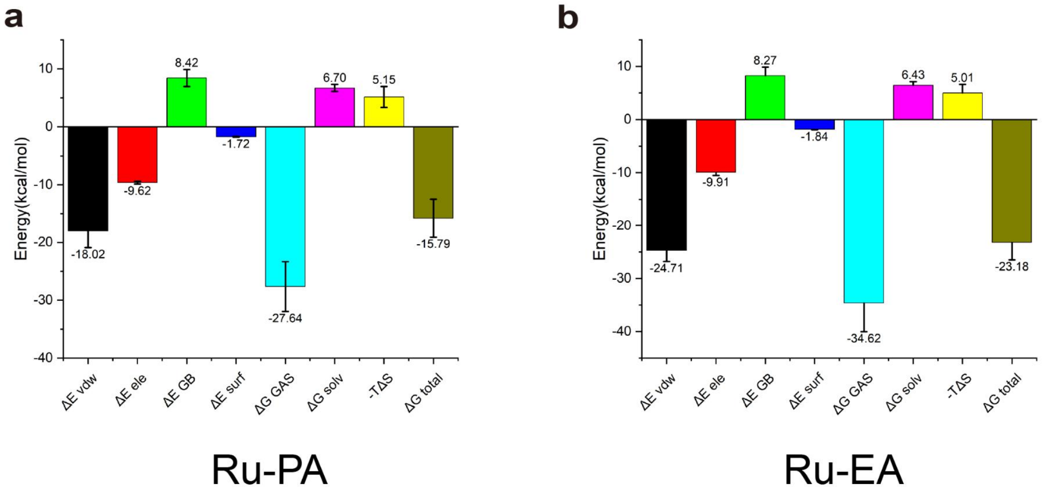

Fig.S17 Comparison of binding free energies of drugs. (a) Ru-PA. (b) Ru-EA.

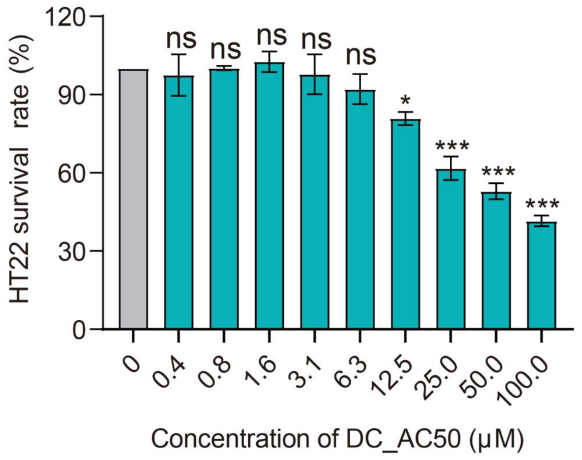

Fig.S18 HT22 cell line toxicity of the ATOX1 protein inhibitor DC_AC50. n=3, $*P < 0.05$, $***P < 0.001$ vs 0 μM.

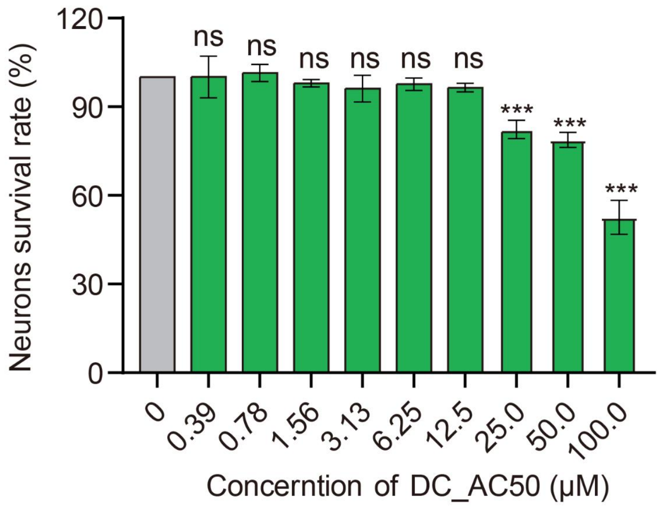

Fig.S19 Neuronal toxicity of the ATOX1 protein inhibitor DC_AC50. n=3, $***P < 0.001$ vs 0 μM.

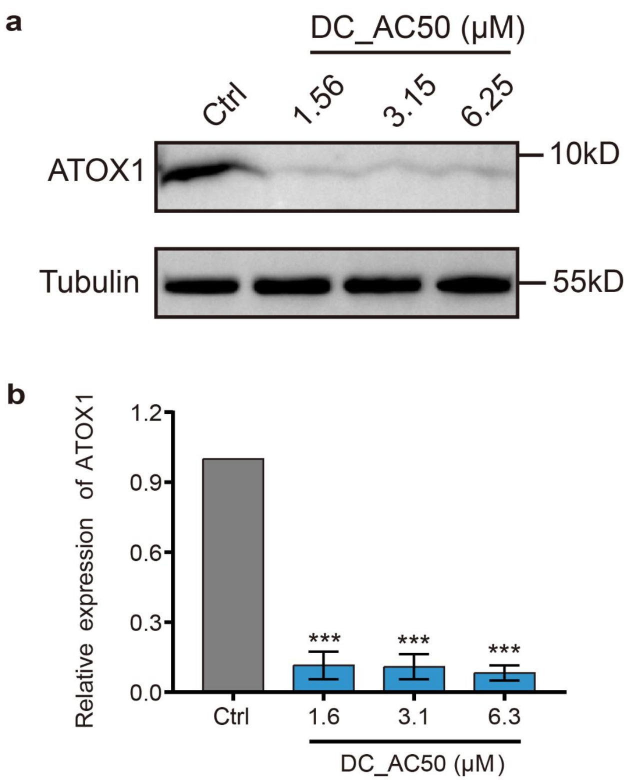

Fig.S20 Inhibition of ATOX1 protein by the inhibitor DC_AC50. (a) Typical plot of Western blotting to detect the effect of different concentrations of DC_AC50 on ATOX1 protein in HT22 cell line. (b) Statistical plot of protein grey values (n=3). ***$P < 0.001$ vs ctrl.

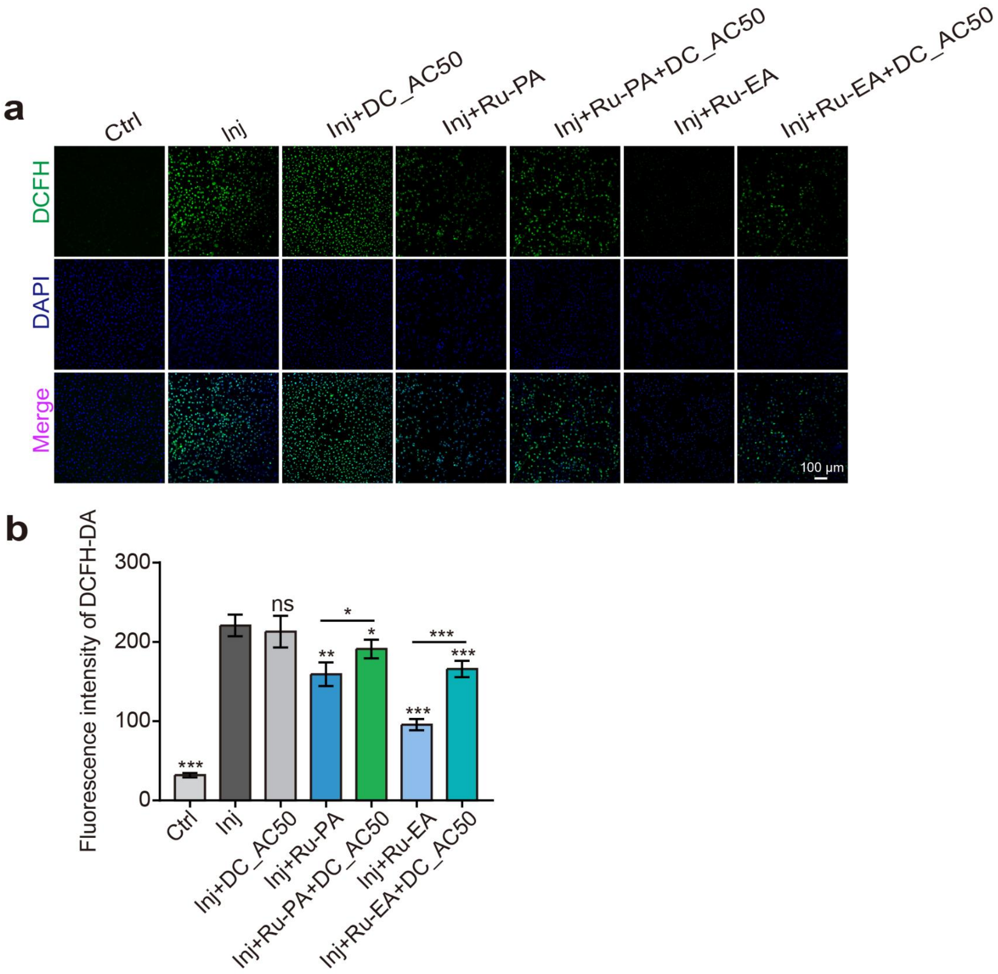

Fig.S21 Changes in drug effects on intracellular ROS levels in HT22 cells after DC_AC50 inhibition of ATOX1 protein. (a) Typical plots of intracellular ROS levels in each group of cells. (b) Statistical plots of intracellular fluorescence levels in each group of cells (n=5). $*P < 0.05$, $**P < 0.01$, $***P < 0.001$.

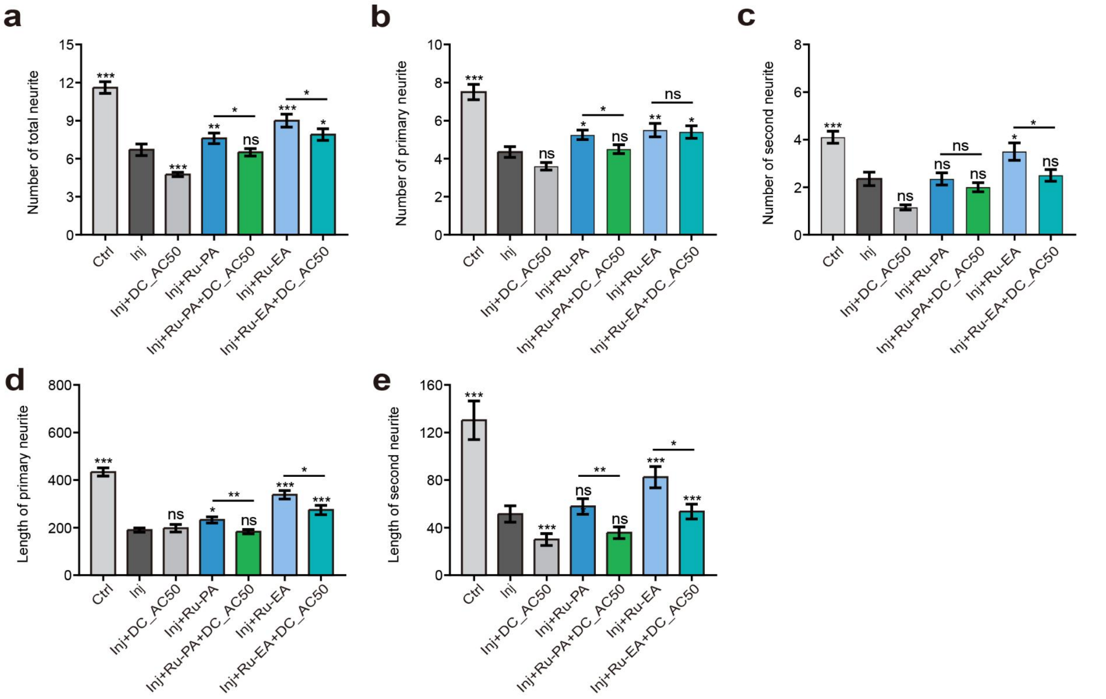

Fig.S22 Changes in the effects of drugs on the length and number of neuronal protrusions after DC_AC50 inhibition of ATOX1 protein. Statistical graph of total number of protrusions (a), number of primary protrusions (b), number of secondary protrusions (c), length of primary protrusions (d) and length of secondary protrusions (e) of neurons (n=20). *$P < 0.05$, **$P < 0.01$, ***$P < 0.001$.
